# Decorating SrTiO_3_ With Cu@Pt Core‐Shell Cocatalysts to Promote the Selective Thermally Assisted Photocatalytic Methanol Dehydrogenation to Formaldehyde

**DOI:** 10.1002/cssc.71077

**Published:** 2026-09-30

**Authors:** Yannik Haver, Zhangjie Zhai, Robert Rinio, Jorik Schaap, Fernando I. Saldaña, Andrea Kirsch, Kasper Wenderich, Bastian Timo Mei

**Affiliations:** ^1^ Laboratory of Industrial Chemistry Ruhr University Bochum Bochum Germany; ^2^ Photocatalytic Synthesis Group Department of Chemical Engineering, Faculty of Science and Technology, MESA+ Institute for Nanotechnology University of Twente Enschede Netherlands; ^3^ Deutsches Elektronen‐Synchrotron DESY Hamburg Germany; ^4^ Research Center Future Energy Materials and Systems of the Research Alliance Ruhr and Faculty of Chemistry and Biochemistry Ruhr University Bochum Bochum Germany

**Keywords:** core–shell, methanol dehydrogenation, operandodiffuse reflectance infrared Fourier transform spectroscopy, photocatalysis, SrTiO_3_

## Abstract

Photocatalytic methanol conversion, a process of particular interest in the context of hydrogen generation, also holds promise as a sustainable approach to chemical synthesis of value‐added products, including formaldehyde. In this study, Cu‐core/Pt‐shell modified SrTiO_3_ photocatalysts are demonstrated to enhance the thermally assisted photocatalytic methanol dehydrogenation in the gas phase by 50% compared to Pt/SrTiO_3_ photocatalysts prepared by conventional photodeposition, while maintaining a high formaldehyde selectivity. Cu‐core/Pt‐shell structures were prepared by means of galvanic displacement, in which Cu is displaced by Pt, yielding irregular core–shell structures consisting of hexagonal Pt particles. These particles transform during extended operation into smooth core–shell systems, thereby enabling stable methanol dehydrogenation. Initial deactivation of the samples is associated with CO‐induced Pt reconstruction, as evidenced by operando diffuse reflectance infrared Fourier transform spectroscopy and transmission electron microscopy. Despite this initial deactivation, the photocatalytic performance is found to be attributable to a significantly enhanced charge separation and carrier lifetime. This enhancement is presumed to be the result of the formation of an Ohmic contact between the SrTiO_3_ absorber and Cu, thereby, enabling efficient charge transfer to the catalytically active Pt surface. This study underscores the beneficial properties of core–shell cocatalysts and highlights the potential for value‐added chemical production using photocatalysis in the gas phase.

## Graphical abstract

Cu‐core/Pt‐shell cocatalysts deposited by galvanic displacement on SrTiO_3_ significantly boost methanol conversion in the thermally assisted photocatalytic methanol dehydrogenation enabling formaldehyde formation along H_2_ production. This enhancement is likely achieved by the formation of Ohmic‐like contacts rather than Schottky junctions.

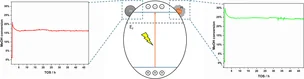

## 1 Introduction

The efficient utilization of solar energy is of critical importance in the effort to effectively combat climate change [1]. Semiconductors (SC) are pivotal materials for solar light utilization due to their capacity to convert light into electrical energy [2, 3]. Still, the inevitable daily and seasonal variations in solar radiation necessitate the development of novel solar energy utilization strategies, in addition to their incorporation into the existing power grid infrastructure. Common alternatives for the utilization of excess energy include the storage of energy in batteries and the transformation into (solar) fuels such as H_2_, as well as the production of valuable chemicals such as formaldehyde [4, 5, 6]. One approach that has received significant attention is the direct utilization of solar light to generate solar fuels or value‐added chemicals through heterogeneous photocatalysis [7, 8]. Heterogeneous photocatalysis is regarded as one of the most straightforward methods for the conversion of solar light to fuels or value‐added chemicals, primarily due to its simplicity [9, 10, 11]. However, the potential of photocatalysis as a cost‐effective technology is yet to be fully realized as its current low light utilization efficiencies require significant enhancement, particularly in photocatalyst design [12, 13].

Recent efforts have focused on leveraging the potential of photocatalysis, particularly in the context of photocatalytic water splitting for hydrogen production, and various strategies have been proposed to enhance efficiency through materials design [12, 14]. The performance evaluation of photocatalysts in the field of water splitting, moreover, frequently involves the utilization of sacrificial agents, such as methanol (MeOH), resulting in enhanced H_2_ production due to the thermodynamically favored MeOH reforming reaction [15, 16]. Despite the current limitations in translating H_2_ production rates obtained by MeOH reforming into overall water splitting, it is crucial to note that the complete oxidation of MeOH also maximizes the formation of CO_2_, the least valuable by‐product of MeOH reforming. Consequently, the selective photocatalytic oxidation of MeOH towards value‐added products has emerged as a subject of interest in recent research. Specifically, the selective oxidation of MeOH to formaldehyde (FA) under both aerobic (Equation  (I)) and anaerobic (Equation‍  (II)) conditions has been reported [17].

(I) $${\mathrm{CH}}_{3}\mathrm{OH}+1/2O_{2}\;\to \;\text{HCHO}+H_{2}O$$

(II) $${\mathrm{CH}}_{3}\mathrm{OH}\;\to \;\text{HCHO}+H_{2}$$

To date, the predominant approach for selective photocatalytic MeOH oxidation involves the use of liquid‐phase suspension reactors, overlooking the potential benefits of conducting such reactions in the gas phase. For instance, a recent study by the Wasserscheid group demonstrates that aerobic thermodynamically downhill MeOH dehydration performed in the gas phase possesses the potential to serve as a cost‐effective alternative to thermal FA processes [18]. Utilizing TiO_2_ (P25) in a small‐scale laboratory setup, production rates of up to 6 mmol min^−1^ g^−1^ FA with a selectivity of ~65% were obtained at elevated temperatures under illumination [18]. Interestingly, Hashimoto et al. [19] reported an 85%–90% selectivity towards methyl formate (MeFo) at room temperature and elevated temperature when using anatase TiO_2_, presumably highlighting the importance of the surface termination of TiO_2_. The aerobic MeOH oxidation results in the stoichiometric formation of water during FA formation [20]. Water formation, however, does not occur during the anaerobic MeOH dehydrogenation reaction. Instead, the process yields water‐free FA, with H_2_ as the coupled product. Thus, anaerobic oxidation eliminates the necessity for costly water separation steps [21] and does not require the implementation of strict safety measures as needed for aerobic oxidation, given that the mixing of MeOH and oxygen can lead to the formation of a potentially flammable and explosive gas mixture [22]. Moreover, the anaerobic oxidation of MeOH facilitates the paired formation of two valuable products, which will potentially lead to an enhancement of the economic viability of such a light‐driven process.

Anaerobic photocatalytic MeOH dehydrogenation showed promising results regarding the FA production in the liquid and gas phase. In the liquid phase, a high apparent quantum yield (AQY) of up to 87 % and production rates of ~8 mmol h^−1^ g^−1^ for FA have been reported over Pd/CdS and Pt/TiO_2_ photocatalysts [23, 24]. In a previous work of our group, Deitermann et al. [25, 26] introduced the continuous thermally assisted anaerobic photocatalytic MeOH conversion in the gas phase over Pt‐loaded SrTiO_3_. A selectivity of up to 75% toward FA was achieved through the implementation of a multi‐parameter optimization process. In addition, trace amounts of CO_2_, CO, MeFo, dimethoxymethane (DMM), and dimethyl ether (DME) were found as byproducts (see Equations (III)–(VI)). These consecutive reactions are believed to proceed over acidic sites, whereas the oxidation reactions require separate oxidation sites [27, 28, 29, 30].

(III) $$\text{HCHO}\;\to \;\text{CO}+H_{2}$$

(IV) $$\text{HCHO }+\;H_{2}O\;\to \;{\mathrm{CO}}_{2}+2H_{2}$$

(V) $$\text{HCHO }+\;{\mathrm{CH}}_{3}\text{OH}\;\to \;\text{HC}O_{2}{\mathrm{CH}}_{3}+H_{2}$$

(VI) $$\text{HCHO }+\;2{\mathrm{CH}}_{3}\mathrm{OH}\;\to \;{\left(H_{3}\mathrm{CO}\right)}_{2}{\mathrm{CH}}_{2}+H_{2}O$$

(VII) $$2{\mathrm{CH}}_{3}\mathrm{OH}\;\to \;H_{3}{\mathrm{COCH}}_{3}+H_{2}O$$

More recently, the anaerobic production of FA with nearly 100% selectivity was observed over Au‐modified F‐etched TiO_2_ [31]. Conversely, we have observed a rather poor selectivity towards FA with equal AQY for MeFo and CO_2_ when using Pt‐modified TiO_2_‐based photocatalysts. In light of the contrasting reports on the aerobic MeOH oxidation process utilizing TiO_2_, and given the established fact that SrTiO_3_ (STO) is recognized as a less acidic material compared to TiO_2_, which in turn imposes limitations on the formation of subsequent reaction products, STO emerges as a promising photocatalyst for selective FA production [32, 33, 34]. STO is known to suffer from high charge carrier recombination rates [35] and usually requires the use of a noble metal cocatalyst (Pt, Rh, and Au) to achieve a reasonable activity and energy conversion efficiency in more demanding reactions like water splitting or MeOH dehydrogenation [36, 37]. The loading of appropriate cocatalysts facilitates charge separation, reduces recombination, and provides active sites for H_2_ evolution [34].

In cocatalyst‐modified photocatalysts, enhanced charge separation is frequently assigned to the trapping of photogenerated electrons achieved by the formation of a Schottky barrier at the noble metal/semiconductor interface. This is due to the high work function of most noble metals known for efficient hydrogen evolution, such as Pt [38, 39, 40]. The resulting upward bending of both conduction (CB) and valance band (VB) at the semiconductor/cocatalyst interface, however, also enables the accumulation of holes at the interface, potentially leading to enhanced recombination (Scheme S1) [41]. In contrast, metallic cocatalysts of lower work function will result in a reduced upward band bending or even a downward band bending, facilitating the desired electron movement towards the cocatalysts [42, 43, 44]. To mitigate Schottky‐induced upward bending and preserve efficient catalysis at the cocatalyst particle, the use of interlayer cores of low‐work‐function materials, such as Cu or Ag, has been proposed. For example, Yu et al. reported the synthesis of Cu@Au/BiVO_4_ photocatalysts to reverse bending at the Cu/BiVO_4_ interface, thereby accelerating the electron transfer to the catalytically active Au shell [45]. Likewise, Chu et al. [46] employed the aforementioned approach for a variety of interlayer materials, concluding that Ag, which has the closest work function to BiVO_4_, resulted in increased activity for photocatalytic H_2_O_2_ generation. A comparable enhancement was reported by Li et al. [47] for g‐C_3_N_4_ photocatalysts. Alignment of the work function of the semiconductor and the cocatalyst, by doping g‐C_3_N_4_ with P, resulted in enhanced water‐splitting performance for CoP/g‐C_3_N_4_. The impact of the formation of Ohmic contacts between STO and cocatalysts has not yet been thoroughly examined, nor has the use of Pt shells to facilitate the HER been investigated.

This study demonstrates that precise tuning of the cocatalyst‐semiconductor junction in Cu@Pt/SrTiO_3_, prepared via galvanic displacement, enables accelerated electron transfer to Pt, enhancing the thermally assisted photocatalytic formaldehyde (FA) production in the gas phase. High‐resolution transmission electron microscopy (HRTEM) and energy‐dispersive X‐ray spectroscopy (EDX) revealed irregular hierarchical structures of platinum (Pt) on copper (Cu). In addition, CO chemisorption and diffuse reflectance infrared Fourier transform spectroscopy (DRIFTS) indicated that Pt is highly dispersed and possesses a high density of strong adsorption sites. Notably, the presented synthesis approach yielded a highly active photocatalyst, achieving a nearly 50% increase in activity and a high FA selectivity. Complementary time‐resolved photoluminescence (TRPL) spectroscopy attributed the enhanced activity to prolonged charge carrier lifetimes. Furthermore, the initial deactivation phenomena were attributed to CO‐induced Pt surface reconstruction, as revealed by in situ DRIFTS analysis of Pt‐CO vibrations. This study offers insights into the long‐term performance of complex photocatalysts and emphasizes the efficacy of Ohmic contacts achieved in core–shell cocatalysts in enhancing the photocatalytic performance of STO‐based photocatalysts.

## 2 Results and Discussion

### 2.1 Photocatalyst Synthesis and Optimization

Common methods for immobilizing cocatalysts on semiconductors include photodeposition (PD) and impregnation, which predominantly result in the immobilization of nanoparticles (NP), but also the formation of core–shell cocatalysts was previously shown as a result of a stepwise photodeposition process [46]. Here, galvanic displacement was used, whereby a less noble metal is substituted for a more noble metal, as demonstrated by Yu et al. [45]. While preparation of a Pt‐loaded SrTiO_3_ sample was conducted in accordance with a well‐established photodeposition procedure, galvanic displacement of Cu by Pt was utilized for the formation of core–shell Cu@Pt cocatalysts [47]. In the present study, 0.1 wt.% Pt on SrTiO_3_ was prepared by photodeposition (PD) and utilized as a benchmark photocatalyst, as our previous work suggested that 0.1 wt.% Pt results in the best performance in MeOH dehydrogenation [25]. For the preparation of core–shell cocatalysts by galvanic displacement (GD), Cu nanoparticles were deposited first on SrTiO_3_ by photodeposition (Scheme S2). Pt shell formation is then achieved by selective deposition atop Cu nanoparticles, which is driven by the potential difference of the relevant Cu and Pt redox couples, i.e., *E* ^0^(Cu^2+^/Cu) = +0.34 V versus NHE versus *E* ^0^(PtCl_6_^2−^/PtCl_4_^2−^) = +0.68 V and *E* ^0^(PtCl_4_^2−^/Pt) = +0.76 V versus NHE [48, 49, 50]. The Cu photodeposition and the subsequent Cu displacement processes were followed by the analysis of the metal concentration in solution using inductively coupled plasma mass spectrometry (ICP‐MS) (see Figure S1). While Cu^2+^ appears to readily adsorb onto STO in the dark, the reduction of Cu during illumination is evident by a color change from white to pale brown during illumination. Successful displacement of Cu by Pt is confirmed by the rapid decolorization of the suspension (white) and the sharp increase in Cu concentration upon the addition of H_2_PtCl_6,_ as revealed by ICP‐MS (Figure S1). The Cu concentration approaches its theoretical maximum for complete Cu dissolution, while the Pt concentration appears to steadily decrease. Various core–shell cocatalyst systems were synthesized by adjusting the Pt:Cu ratio used during galvanic displacement in the range of 2:1 to 10:1. The corresponding samples were named by the amount of the precursor and the synthesis method utilized. Thus, 04Pt_01Cu_GD refers to a sample that was synthesized by galvanic displacement (GD) aiming at a loading of 0.4 wt.% of Pt and 0.1 wt.% of Cu. 01Pt_PD refers to the benchmark photocatalyst prepared by photodeposition (PD) aiming at a loading of 0.1 wt.% of Pt on STO.

Despite the evident changes in color, the deposition of Cu and Pt was confirmed through additional elemental analysis using ICP‐MS. A lower platinum (Pt) loading was observed for all GD samples in comparison to the PD sample, even though a higher amount of the Pt precursor was utilized during the GD synthesis (Table 1). Importantly, the determined amount of Pt for samples prepared by galvanic displacement only deviates slightly from the previously determined optimum of 0.1 wt.% Pt [25]. These low loadings are also in good agreement with common observations in photocatalysis, i.e., a rather low amount of cocatalyst results in the best photocatalytic activity, which can be explained, among other factors, by the EPAO model proposed by Bowker et al. [51].

**TABLE 1:** Results of catalyst characterization by means of ICP‐MS (Sr, Ti, Pt, Cu content), CO chemisorption (Pt dispersion), bandgap (UV–vis), and TRPLS (half live times of photoluminescence).

Sample	Elemental Analysis (ICP‐MS)		Chemisorbed	**Bandgap** **(Tauc method)**	TRPL lifetimes		
	Pt, wt.%	Cu, wt.%	CO, nmol g_Pt_^−1^	eV	*τ*_1_, ps	*τ*_2_, ps	*τ*_3_, ns
01Pt_PD	0.16	<0.05	279	3.23	35.1	210	0.93
02Pt_01Cu_GD	0.03	<0.05	1134	3.18	—	—	—
03Pt_01Cu_GD	0.08	<0.05	910	3.21	55.4	305	1.5
04Pt_01Cu_GD	0.09	<0.05	791	3.22	68.8	527	3.9

All photocatalyst samples were tested for their performance in MeOH dehydrogenation after immobilization by spray coating (Table S1) as thin films (1–1.3 mg cm^−2^) on a glass plate. Before performance evaluation, the immobilized thin films were pretreated under illumination for 18 h with 365 nm LEDs (52.9 mW cm^−2^) in a moist N_2_ stream (50 ml min^−1^, 4.5% H_2_O). Subsequently, the materials were tested under identical reaction conditions (see Table S2, balancing conversion, FA yield, and stability) in the flat‐plate reactor (Figure S2) operated at 100 °C using a continuous reactant gas feed.

As shown in Figure 1A, the 04Pt_01Cu_GD sample exhibited the highest MeOH conversion, reaching 24%, which signifies a 50% enhancement over the Pt PD reference sample (16%). Notably, the selectivity to FA was retained at 65% for 04Pt_01Cu_GD, corresponding to an FA production rate of 4.9 µmol/h/cm^2^ or 3200 µmol/h/g (Figure S3). However, comparison of mass‐normalized production rates requires caution. 02Pt_01Cu_GD was tested at lower catalyst loading and consequently exhibited a higher production rate when normalized to catalyst mass. Nevertheless, the catalyst‐loading series shown in Figure S4 demonstrates that the activity does not scale linearly with the catalyst loading. Increasing the catalyst loading increases the amount of catalyst available for reaction but also changes the optical thickness, gas–solid contact time, and extent of secondary reactions. Consequently, catalyst mass‐normalized rates alone do not provide an unambiguous measure of photocatalyst activity in the present flat‐plate reactor. For this reactor configuration, the illuminated area and the catalyst loading per illuminated area are therefore important parameters for evaluating reactor‐level productivity. The areal production rate is particularly relevant for comparing immobilized photocatalyst films, whereas catalyst‐mass‐normalized rates remain useful for comparison with literature reports. To account for different Pt contents of the sample, we additionally normalized the H_2_ and FA production rates to the Pt mass determined by ICP‐MS and to the accessible Pt surface areas estimated from CO chemisorption (Figure S5). On both Pt‐based normalization bases, 02Pt_01Cu_GD exhibited the highest apparent specific activity. In contrast, 04Pt_01Cu_GD represented the best overall catalyst when reactor‐level productivity and selectivity were considered. The apparent volcano‐shape dependence of conversion on the nominal Pt:Cu ratio likely results from competing effects of cocatalyst coverage, Pt‐Cu interfacial contact, particle growth, and cocatalyst distribution. Because the Pt:Cu ratio was varied by changing the amount of Pt precursor while keeping the Cu deposition conditions largely constant, the resulting samples differ not only in Pt loading but also in the extent of galvanically displaced Cu, Pt particle size, Pt coverage, and the amount of the Pt‐Cu interface. The activity cannot therefore be attributed to the nominal Pt:Cu ratio alone. This behavior is qualitatively consistent with recent calculations and the EPAO model [51, 52], in which increasing cocatalyst coverage initially expands the photocatalytically promoted area, whereas excessive coverage or particle growth can lead to an overlap of these regions and a decrease in effective activity. We therefore hypothesize that 04Pt_01Cu_GD provides a favorable balance between cocatalyst coverage, Pt‐Cu interfacial contact, and particle size, whereas further Pt or Cu addition may promote cocatalyst growth and reduce the effective interfacial area. The structural basis of this interpretation is examined in the subsequent catalyst‐characterization section.

**FIGURE 1:**
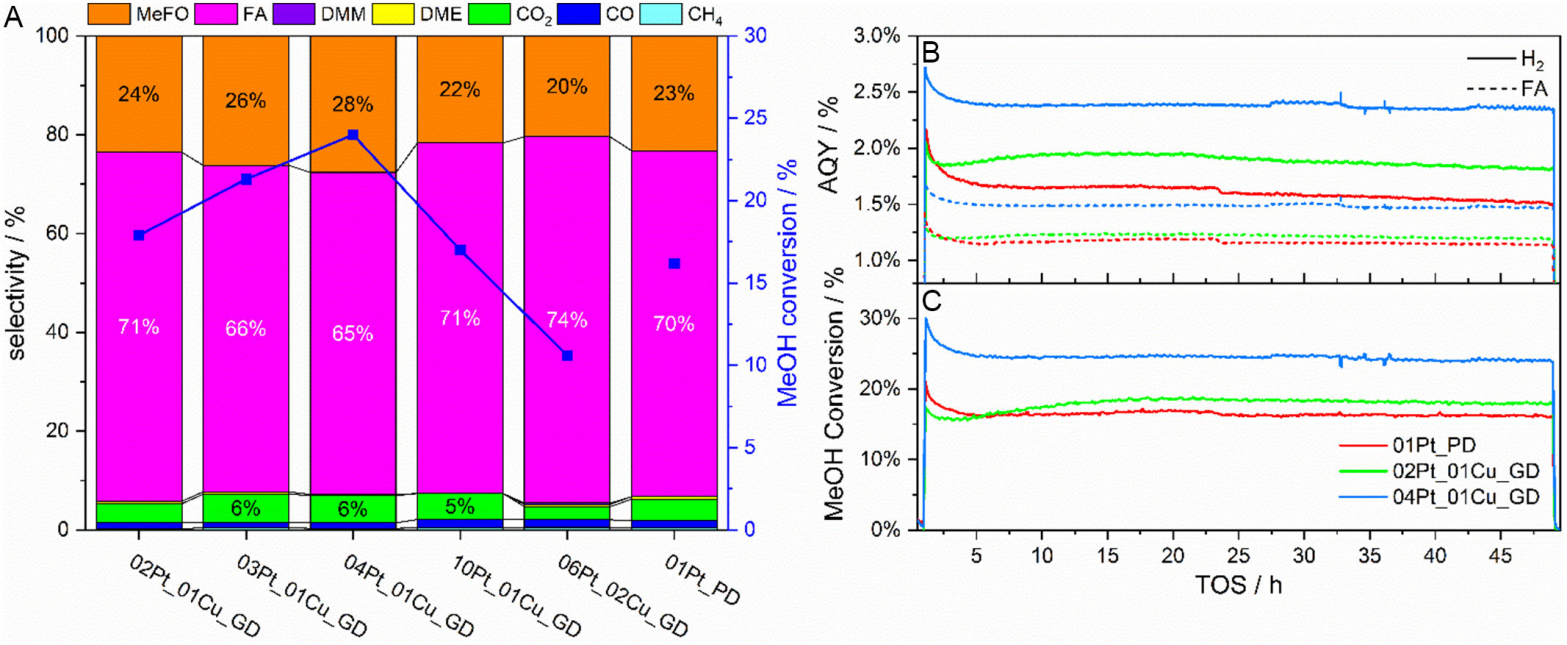
(A) MeOH conversion and selectivity to CO_2_ (green), CO (blue), formaldehyde (FA, pink), methyl formate (MeFo, orange), and trace components (DMM, dimethoxy methane in purple, DME, dimethyl ether in yellow, and CH_4_ in cyan) for *x*Pt_*y*Cu_GD (*x* = 02–10, *y* = 01−02) and 01Pt_PD during thermally assisted anaerobic photocatalytic MeOH dehydrogenation in the gas phase at *T* = 100 °C. Conversion and selectivity were determined after 47 h of continuous photocatalytic reaction. (B) Apparent quantum yield for H_2_ and FA versus TOS. (C) MeOH conversion versus time on stream (TOS).

However, product distribution was only affected to a minor extent. At higher conversions (11% to 24%), the selectivity toward FA decreased (74% to 65%), while the MeFo yield (20% to 28%) and the CO_2_ yield (3% to 6%) increased accordingly. Deitermann et al. [25] previously observed a similar trend when tuning conversion via light intensity. They found that increasing conversion decreased FA selectivity while increasing MeFo selectivity. The higher CO_2_ and MeFo selectivity at higher conversion can be explained by efficient charge separation and presumably inefficient product transport, which leads to overoxidation to MeFo and CO_2_, both of which require additional charges (holes) to be transferred to the adsorbed intermediate for their formation. While this implies that utilizing core–shell systems with an optimized Pt:Cu ratio results in a more efficient photocatalyst, the general surface reaction mechanism involving MeOH adsorption at the STO surface is unaffected by cocatalyst modifications. Furthermore, the data suggest that improving mass transport by shortening the contact time between the substrate and the photocatalyst could enhance FA selectivity. A mass loading variation also supports this assumption, revealing that, as the photocatalyst loading increases from 1.23 to 7.16 mg cm^‐2^, conversion steadily increases, along with higher selectivity toward DMM and MeFo formation (Figure S4). Consequently, an increase in mass loading results in an increase in the contact time between the substrate and the photocatalyst, leading to overoxidation.

The MeOH conversion during 48 h time on stream (TOS), along with the AQY, are shown for GD and the benchmark PD samples in Figure 1B. The AQY for H_2_ is generally higher than that for FA since H_2_ evolution is the only reductive reaction occurring in the system, while some of the photogenerated holes are also involved in overoxidation. Thus, the AQY of H_2_ is an indicator of the overall light‐harvesting efficiency, whereas the AQY of FA is an indicator of the selective utilization of light for the desired product. A decrease in the AQY values and MeOH conversion under illumination during the first 5 h of TOS reveals significant deactivation for all samples. Interestingly, after initial deactivation, the 02Pt_01Cu_GD sample appears to reactivate, while a minor, slow decrease is observed after the initial rapid deactivation for the 01Pt_PD and 04Pt_01Cu_GD samples. Furthermore, a more pronounced activation was observed for 03Pt_01Cu_GD (see Figure S6). Nevertheless, 04Pt_01Cu_GD’s performance after 48 h of TOS was still superior. The deactivation and subsequent activation behaviors are likely indicative of a change in the photocatalyst, which will be addressed and discussed in detail below.

Importantly, the photocatalyst performance evaluation revealed the superior behavior of the core–shell cocatalyst samples prepared by GD. The initial AQY determined from the H_2_ evolution was 2.72% for 04Pt_01Cu_GD, with a 14% deactivation to 2.35% after 48 h of TOS. A similar deactivation of 12% in AQY_H2_ was observed for 02Pt_01Cu_GD, which started from an initial AQY of 2.06%. Likewise, AQY_FA_ decreased by 13% and 12%, respectively (Table S4). The improvement in stability is particularly evident when comparing to the PD sample. For the PD sample, a deactivation of 31%, corresponding to a decrease in AQY_H2_ from 2.16% to 1.5% after 48 h of TOS alongside a decrease in AQY_FA_ by 21%, was revealed. Overall, the optimized 04Pt_01Cu_GD demonstrates a comparable MeOH conversion as the Pt/TiO_2_ samples yet exhibits a significantly superior selectivity for FA formation (see Figure S7). Furthermore, these findings underscore the necessity of employing long‐term measurements to ensure the reliable evaluation of catalyst activity and stability. A systematic kinetic analysis involving variation of MeOH and water partial pressures, light intensity, and reaction temperature was beyond the scope of the present study. The influence of these reaction parameters on thermally assisted gas‐phase MeOH conversion over Pt/SrTiO_3_ was investigated in our previous work, while the corresponding reaction mechanism was addressed separately [25, 26]. These studies provide the kinetic and mechanistic basis for the parent Pt/SrTiO_3_ system. In the present work, the focus is placed on the effect of the Cu@Pt cocatalyst structure on charge‐carrier dynamics, Pt surface properties, selectivity, and long‐term stability. A direct comparison of apparent kinetic parameters between the different Cu@Pt samples would additionally require careful control of catalyst loading, optical thickness, mass transfer, and dynamic surface restructuring.

A general comparison of the performance of 04Pt_01Cu_GD with other materials reported in the literature is challenging due to the unique nature of the fixed‐bed gas‐phase reactor and the reaction conditions employed in this work. Photocatalytic MeOH dehydrogenation is typically reported using photocatalyst suspensions in liquid‐phase batch reactors. In such systems, high AQYs for formaldehyde of up to 87% have been achieved using CdS or TiO_2_‐based photocatalysts [23, 24]. Nonetheless, the measurement of these high AQYs is typically conducted in relatively small setups under conditions of intense illumination, and the durability of these materials is typically not well documented. Moreover, translating discontinuous liquid‐phase processes into continuous flow processes is a significant challenge, which limits the scalability of these systems to industrial standards.

Comparable studies on photocatalytic gas‐phase MeOH dehydrogenation are scarce. Wasserscheid et al. [18] employed a similar gas‐phase reactor for aerobic MeOH oxidation to FA over TiO_2_ P25, achieving production rates ranging from 6 to 0.5 mmol FA min^−1^ g^−1^_Cat_ at catalyst loadings of 0.3–4 mg cm^−2^. The maximum AQY for FA was 7.1% under optimized conditions. While this work generally demonstrates the potential of a gas‐phase reactor geometry, it particularly highlights the significant impact of the thermodynamic driving force provided by oxygen on quantum efficiency. Still, aerobic MeOH oxidation results in larger costs associated with product separation and process safety risks due to the explosive nature of the gas mixtures. Thus, anaerobic MeOH dehydrogenation could potentially surpass the economic viability of aerobic MeOH oxidation even at a lower conversion [18]. The very recent work of Kandiel et al. [31] offers the most comparable study to our research by performing thermo‐assisted photocatalytic MeOH dehydrogenation over Au‐loaded fluoride‐etched TiO_2_ spheres. While the catalyst synthesis appears to be rather complex, the authors report that only hydrogen (H_2_) and FA are obtained at mild reaction temperatures of 105 °C, with a production rate of 1800 µmol h^−1^ g^−1^ H_2_ at a maximum AQY of 7.1% for H_2_. Although the AQYs for FA were not reported in the study, the authors claimed a roughly 1:1 ratio of H_2_ to FA production. Given that performing MeOH dehydrogenation over Pt on TiO_2_ photocatalysts using our reactor at similar reaction conditions resulted in a strong deactivation, as apparent by the decrease in AQY_H2_ from 14.5% to 5.5 % within 48 h (Figure S7) and a significant formation of CO_2_ and MeFo, the long‐term durability is still to be revealed for Au‐loaded fluoride‐etched TiO_2_ spheres proposed by Kandiel et al. [31]. Clearly, a thorough comparison of different catalysts requires rigorous testing using identical reactor designs and conditions as values such as AQY and production rate normalized to catalyst mass are still significantly influenced by light intensity, illuminated area, and catalyst mass per area. Therefore, notwithstanding the challenges associated with comparison to alternative systems, the tunable activity, high selectivity, and exceptional stability of SrTiO_3_ achieved by cocatalysts design renders the system well‐suited for continuous, and presumably even industrial‐like, operation where longevity is crucial and would likely be regarded as more significant than peak activity.

### 2.2 Detailed Catalyst Characterization

To confirm the successful synthesis of Cu‐core/Pt‐shell cocatalysts, to derive a structure‐performance relation, and rationalize the volcano‐shaped activity trend discussed above, the materials were analyzed using complementary microscopy techniques (SEM, TEM, and STEM), CO chemisorption, CO DRIFTS, UV–vis, and ICP‐MS to characterize and quantify different Pt and Cu sites. Moreover, detailed time‐resolved photoluminescence (TRPL) measurements were performed to deduce the impact of the different cocatalysts (PD vs. GD) on the charge separation efficiency.

The Pt dispersion was analyzed using CO chemisorption (Table 1), assuming that CO is predominantly adsorbing on Pt [53]. According to the Pt dispersion, it is evident that the activity of the GD samples is closely related to a decrease in available Pt surface sites for the samples evaluated. Although the general activity‐dispersion trend for the GD samples holds (Figure S5), with the lowest CO amount of 791 nmol g^−1^ being determined for 04Pt_01Cu_GD, comparison of the different GD samples with the 01Pt_PD sample suggests that the available Pt surface only partially explains the activity trend. The 01Pt_PD sample clearly possesses the lowest dispersion (279 nmol g_Pt_^−1^) of all samples tested, but even the sample with the highest dispersion (02Pt_01Cu_GD) was shown to possess superior activity. Thus, based on the CO chemisorption and ICP analysis, it can be concluded that Pt dispersion is only partly correlated with photocatalytic activity for MeOH dehydrogenation. Instead, the data hint at a general positive effect of Cu.

Complementary microscopy techniques were used to further confirm the presence of Cu and the formation of Cu‐core/Pt‐shell cocatalysts. Scanning electron microscopy (Figure S8) revealed the presence of irregular cocatalyst particles with a diameter of ~10 nm on SrTiO_3_ for both 03Pt_01Cu_GD and 04Pt_01Cu_GD. These particles contain a central core with protruding spikes, as has been observed previously, for example, for NiS@Au/g‐C_3_N_4_ [54]. With increasing Cu and Pt loading, as in 06Pt_02Cu_GD, larger ~20–30 nm islands appear. In contrast, 01Pt_PD contains small (2–4 nm) and relatively uniform nanoparticles, as commonly obtained by photodeposition [55, 56]. The larger cocatalyst islands observed for 06Pt_02Cu_GD may reduce the accessible SrTiO_3_ cocatalyst interfacial area and promote overlap of the photocatalytically promoted regions, as described qualitatively by the EPAO model, contributing to the lower conversion observed for this sample. Because the particle‐size and interfacial‐area distributions were not quantitatively determined for all samples, this interpretation remains a mechanistic hypothesis rather than definitive proof. Further characterization of the 04Pt_01Cu_GD using high‐resolution TEM (Figure 2A,C) highlighted the presence of hexagonal Pt nanoparticles assembling into larger structures. Lattice fringes of 0.202 and 0.232 nm match Pt(200) and Pt(111), respectively, consistent with a truncated octahedral Pt morphology [57, 58, 59]. Importantly, EDS line scans revealed that the EDS signal corresponding to Pt is observed over a wider range than that of the EDS signal corresponding to Cu (Figure 2B,D). The round NPs observed for 01Pt_PD (Figure 2E–F) were shown to consist entirely of Pt. Importantly, EDS analysis resulted in the detection of Pt only (Figure 2H), thereby ruling out cross‐sensitivity in the EDS analysis. Identical qualitative Pt/Cu line‐scan profiles were also observed for 03Pt_01Cu_GD and 06Pt_02Cu_GD (Figure S9). Because of the low total cocatalyst loading and heterogeneous particle distribution, the EDS data do not provide a statistically representative local Cu/Pt ratio or a precise average shell thickness. The shell thickness inferred from individual line scans should therefore be regarded as a local estimate rather than a quantitative average. To obtain complementary information about the average local structure and Cu/Pt incorporation, total scattering experiments followed by pair distribution function (PDF) analysis were performed. The total scattering pattern and corresponding PDF of 04Pt_01Cu_GD do not show a distinct Cu@Pt contribution because of the low cocatalyst concentration and the dominant scattering contribution from the SrTiO_3_ support (Figure S10). However, subtraction of the PDF of untreated SrTiO_3_ from that of 04Pt_01Cu_GD reveals an fcc metallic contribution in the difference PDF (Figure S11). The difference PDF is better described by Cu‐containing fcc Pt models than by pure fcc Pt or pure fcc Cu, with the CuPt_7_ model providing the closest agreement. Refinement of this model yields a lattice parameter of 7.7536 Å. Comparison with reference structures and a Vegard‐law calibration gives an estimated Cu content of approximately 20% relative to Pt in the fcc metallic phase (Figure S12) [60]. These results support the presence of a Pt‐rich metallic Cu‐Pt alloy or intermixed Pt‐Cu region within the cocatalyst. The totalscattering data therefore provide complementary evidence that Cu is present at least partly in a metallic, zero‐valent state and that Pt‐Cu intermixing occurs at the atomic scale. However, the PDF analysis does not resolve the spatial arrangement of this alloyed region relative to the SrTiO_3_ support and thus does not independently prove a uniform core–shell geometry. We therefore describe the structure as a Cu‐containing, Pt‐rich core–shell/intermixed cocatalyst, with the macroscopic core–shell morphology supported by TEM/EDS and the local metallic Pt‐Cu character supported by PDF analysis.

**FIGURE 2:**
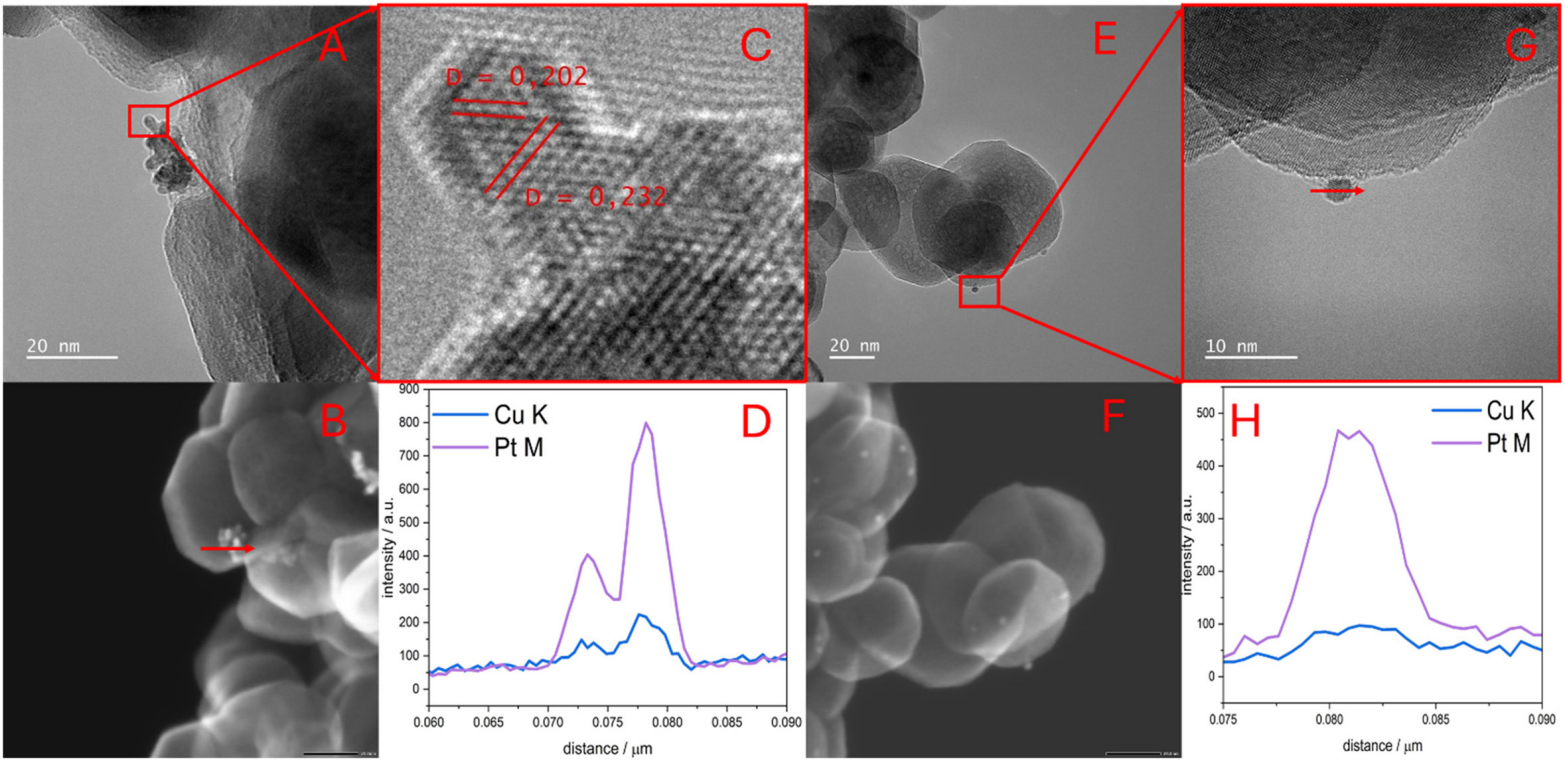
(A–D) TEM/STEM and EDS line scan of the 04Pt_01Cu_GD photocatalyst, evidencing Cu‐core/Pt‐shell structures. For (E–H) 01Pt_PD rounded Pt NPs are observed and (H) only Pt is revealed in the corresponding EDS line scan. Scale bars as shown.

For 02Pt_01Cu_GD, a distinct core–shell structure could not be resolved, most likely because of the low Pt loading and the resulting weak Pt signal. Taken together, the microscopy, ICP‐MS, and total scattering results support the proposed structure‐performance relationship: the activity depends not only on total Pt and Cu loading, but also on cocatalyst particle size, Pt coverage, Pt‐Cu interfacial contact, and the extent of atomic‐scale Pt‐Cu intermixing. In particular, the larger cocatalyst islands observed for 06Pt_02Cu_GD provide a possible structural explanation for its lower conversion.

Subsequent characterization of the cocatalyst particles was conducted using DRIFTS with CO as a probe molecule to identify distinct Pt sites within the catalyst. This approach was employed to overcome the limitations of electron microscopy, which provides only spatially localized information. CO‐DRIFTS (1000 ppm CO/N_2_,10 mL/min, 35 °C) was performed after oxidative cleaning (syn. air, 10 mL/min, 200 °C, 1 h) and subsequent reduction (200 °C,10 mL/min, 1 h) of the samples to establish a reproducible surface state for CO adsorption. For the 01Pt_PD sample, a single broad Pt‐CO band (*ν*_max_ = 2082 cm^−1^) is observed (Figure 3A) [61, 62, 63, 64, 65, 66, 67, 68], while for the GD samples, an additional high‐wavenumber band (2120 cm^−1^) appeared, which can be assigned to a Cu‐CO vibration (Figures 3B,C and S13) [69]. Besides the appearance of the additional high‐wavenumber peak, major differences between the samples are noticed in the overall CO band intensity, which is in general agreement with the amount of Pt. As such, the overall intensity follows the Pt loading from ICP‐MS (01Pt_PD, highest total intensity, 02Pt_01Cu_GD, lowest total intensity). This may appear to contradict expectations given the common assumption that the Pt dispersion is the predominant factor influencing the intensity of the Pt‐CO band, superseding the total quantity of Pt. However, it should be noted that the IR intensity in DRIFTS is also affected by the color of the samples. Consequently, a direct comparison of total intensities might not provide a reliable assessment. The intensity of the Cu‐CO band clearly follows the Pt loading, and the Cu‐CO band decreased with higher Pt loading (Figure S13). The Cu‐CO contribution is substantially stronger for 06Pt_02Cu_GD than for 04Pt_01Cu_GD. This difference is consistent with the lower nominal Pt:Cu ratio of 06Pt_02Cu_GD, which likely results in less complete Pt coverage of the Cu‐containing cocatalyst and a greater number of Cu sites accessible for CO adsorption. In contrast, the weak Cu‐CO contribution for 04Pt_01Cu_GD is consistent with a comparatively thick Pt shell, as also suggested by the absence of a detectable Cu signal in the XPS survey spectrum of this sample (Figure S14). The Cu‐CO intensity should nevertheless not be interpreted as a direct quantitative measure of the total Cu content. Because DRIFTS measures changes in diffuse reflectance, the signal intensity is affected by the sample’s reflectivity, color, morphology, particle size, optical properties, and spectral baseline in addition to the amount of adsorbed CO. The stronger Cu‐CO band for 06Pt_02Cu_GD therefore indicates a higher relative accessibility of Cu sites under the measurement conditions rather than a quantitatively proportional increase in the total amount of Cu. From the UV–vis spectra (Figure S15) and the corresponding bandgaps (Table 1), which were determined using the Tauc method, it can be concluded that there has been no change in the bulk properties of the SrTiO_3_ and that an localized surface plasmon resonance (LSPR) effect of the Cu, due to photodeposition or galvanic displacement, is also unlikely since no significant change compared to the starting material was observed.

**FIGURE 3:**
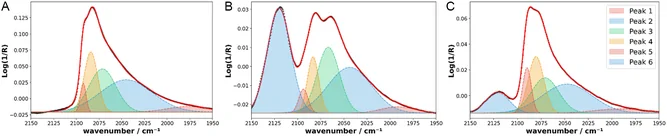
DRIFTS obtained after exposure of the photocatalyst for 15 min to a 1000 ppm CO/N_2_ stream (35 °C, blank‐subtracted): (A) 01Pt_PD, (B) 02Pt_01Cu_GD, (C) 04Pt_01Cu_GD.

Further insights were obtained by deconvoluting the Pt‐CO band following a model for 2.3 nm Pt truncated octahedron nanoparticles reported by Cheah et al. [61]. The model specifically considers CO interaction with Pt in different coordination environments (see also the assignment of the peaks summarized in Table 2), which thereby allows differentiation between Pt in a low and high‐coordination environment. For 04Pt_01Cu_GD, a balanced ratio of the peaks assigned to low‐coordinated Pt sites (LC, peaks 2+3) and high coordinated Pt sites (HC, peak 4 + 5) was revealed (Figure 3C). Moreover, the peak intensities of peaks 4 and 5 were found to be rather similar, reflecting a balanced ratio of HC Pt sites in (111) and (100) crystal facets, which is in agreement with the truncated octahedral structure observed by TEM. In contrast, peak 4 is found to be significantly more intense for 01Pt_PD, indicating that the (111) facet is dominating. This deviation from the truncated octahedral structure is also in line with the rounder NP shape observed by TEM. Finally, for the 02Pt_01Cu_GD sample, low coordinated Pt is dominating, as revealed by the rather intense peaks 2 and 3. This suggests that the Pt surface in 02Pt_01Cu_GD is dominated by kink and edge sites. Overall, the deconvolution using the peak model clearly revealed differences in the Pt surface structure. Moreover, TEM, EDS, and CO‐DRIFTS analysis collectively proved the formation of core–shell cocatalysts and highlighted distinct difference between cocatalyst structures deposited by PD and GD in shape and surface termination. While PD resulted in small, round NP with the Pt (111) facet dominating, GD synthesis resulted in larger Cu‐core Pt‐shell structures with distinct features of truncated octahedral Pt crystallites, with the degree of Cu coverage depending on the Pt amount.

**TABLE 2:** Fitting parameters and peak assignment for the deconvolution of Pt/Cu‐CO band. The peak assignment was adapted from [61].

	Designation	***ν*(CO), ±10 cm** ^**−1**^	**FWHM, cm** ^**−1**^
Peak 1	Bridge bonded CO	1975	50
Peak 2	Ultra‐low coordination Pt (kinks)	2030	65
Peak 3	Low coordination Pt (edges)	2067	30
Peak 4	High coordination Pt (111)	2075	16
Peak 5	High coordination Pt (100)	2080	8
Peak 6	CO‐Cu	2120	16

To investigate the charge carrier dynamics and evaluate the charge separation efficiency of the prepared photocatalysts, picosecond time‐resolved photoluminescence (TRPL) spectroscopy was performed (excitation at 266 nm). The photoemission behavior revealed clear trends regarding the role of the cocatalysts. For the 01Pt_PD sample, the highest photoluminescence intensity is observed (Figure 4A). For Cu‐containing samples, the emission intensity is significantly quenched and further decreased with increasing Pt loading. This quenching is accompanied by a blueshift of the emission peak (Figure 4B). The PL quenching thus indicates that copper acts as an effective electron sink, facilitating rapid electron transfer from the STO conduction band to the metal and fundamentally suppressing radiative electron–hole recombination. Similar trends have been observed previously in TiO_2_ loaded with Pt [70], Cu or Fe [71], and in g‐C_3_N_4_ loaded with Pt nanoparticles [72].

**FIGURE 4:**
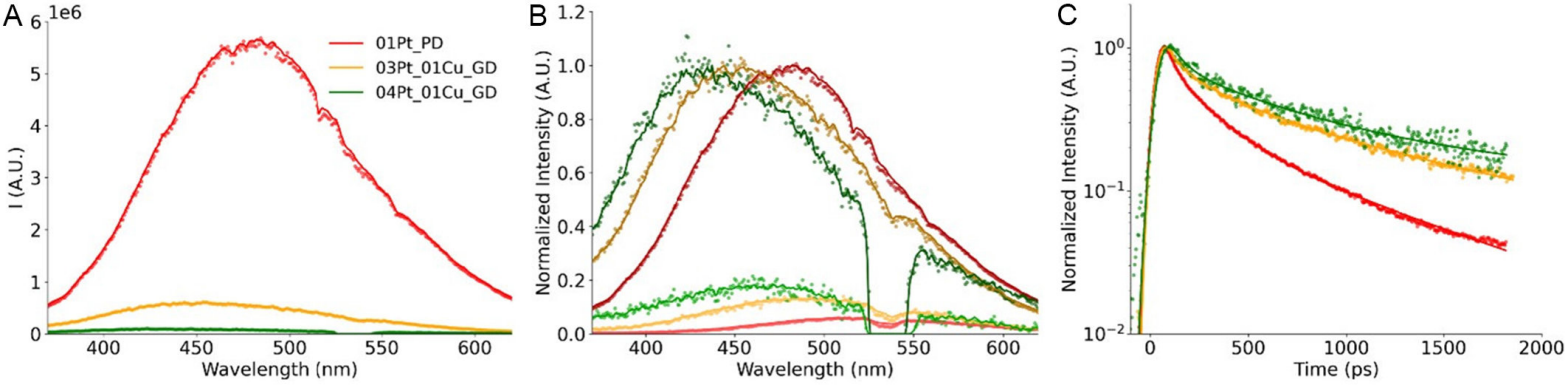
(A) Photoluminescence intensity at 100 ps. Data captured using the same acquisition parameters (Excitation at 267 nm and 100 uW intensity, detector gain 63, Integration time 500 s), (B) dark colors spectrum at 100 ps and lighter colors spectrum at 1500 ps. (C) Time traces at 500 nm. Points are the raw data and lines the fits obtained from global analysis. The dip at 530–550 nm is due to residual scattering of the excitation laser.

The spectral time evolution of the PL signal was followed to further understand charge carrier recombination. In all samples, a redshift of the PL over the course of the experiment was observed (Figure S16). This temporal shift is characteristic of charge carrier relaxation in semiconductors, indicating the spatial migration of electrons and/or holes from shallow bulk states toward deeper trap states at the nanoparticle surface [73, 74, 75, 76]. To quantify the spectral shifts of the PL, the PL spectrum at 100 ps was fitted using a Gaussian, from which the emission value was approximated. For the 01Pt_PD sample, an emission peak around 485 nm was obtained (see Figure 4B), while for the GD samples, the emission center undergoes a blueshift to 462 nm (03Pt_C01Cu_GD) and 445 nm (04Pt_C01Cu_GD). This optical shift of up to 39 nm (~0.23 eV) suggests that for samples containing copper, low‐energy electrons from deep trap states are preferentially scavenged or that copper effectively passivated surface defect states responsible for longer‐wavelength radiative recombination. Consequently, the remaining radiative pathways are dominated by higher‐energy, shallow‐state transitions.

To further quantify the charge carrier migration, the TRPL decay curves were fitted using a three‐component parallel model via global analysis. The model components are attributed to three distinct processes: bulk recombination within the SrTiO_3_ shortly after photoexcitation (*τ*_1_); trapping and recombination of charge carriers at the surface or at the cocatalyst (*τ*_2_), which, in SrTiO_3_, are known to occur at the surface within ps timescales in the form of oxyl radicals on (Ti^3+^) [77] and also have been shown to migrate to Pt on Pt‐loaded SrTiO_3_ [78], and the delayed recombination of surface‐trapped holes with electrons (*τ*_3_). As summarized in Figures 4C, S17 and Table 1, for GD samples, a prolonged PL lifetime for all values of *τ* is observed, with the prolongation being most significant for *τ*_3_, followed by *τ*_2_ and then *τ*_1_. This lifetime enhancement indicates that charge separation is significantly enhanced compared to that of the 01Pt_PD sample. Particularly, the longer bulk recombination time (*τ*_1_) together with the strong PL quenching observed for GD samples suggests that the presence of copper significantly improves electron–hole separation in the bulk. The extended carrier lifetimes of *τ*_2_ and *τ*_3_ further suggest that rapid (400 ps) hole migration to the metal, caused by the formation of a Schottky barrier at the metal semiconductor interface [78], which also partially results in Pt acting as a recombination center, is efficiently suppressed. Thus, the utilization of Cu‐core/Pt‐shell cocatalysts resulted in clear enhancements in charge separation, with recombination losses also being mitigated, which is in excellent agreement with the enhanced macroscopic catalytic performance of the samples prepared by GD in MeOH dehydrogenation.

Overall, the combined characterization results support a structure‐performance relationship in which the activity of the Cu@Pt/SrTiO_3_ photocatalysts is governed by cocatalyst morphology, Pt‐Cu interfacial contact, Pt surface structure, and charge‐carrier dynamics rather than by the total Pt or Cu loading alone. TEM, STEM, SEM, EDS, and PDF analyses support the formation of Cu‐core/Pt‐shell cocatalysts, while also revealing larger 20–30 nm cocatalyst islands for 06Pt_02Cu_GD compared with the approximately 10 nm structures observed for 03Pt_01Cu_GD and 04Pt_01Cu_GD. These larger islands may reduce the effective SrTiO_3_ cocatalyst interfacial area and contribute to the lower conversion of 06Pt_02Cu_GD. The TRPL results show more efficient charge separation for the GD samples, as indicated by PL quenching and prolonged fitted carrier lifetimes. The UV–vis measurements reveal no significant change in the SrTiO_3_ optical bandgap, arguing against substantial bulk Cu doping as the origin of the observed photophysical differences. Although metallic Cu is formed during photodeposition and PDF analysis clearly supported the presence of metallic Cu, partially oxidized Cu species at the Cu‐SrTiO_3_ interface cannot be fully excluded but appear highly unlikely. Such species may contribute to an interfacial dipole and modify the effective work function and band alignment. In addition, electronic equilibration across the Cu‐Pt interface may increase the electron density of the Pt shell without necessarily producing a detectable change in the formal oxidation state of metallic Pt. The differences observed in the CO‐DRIFTS spectra further indicate that the Pt surface environment is modified in the GD samples. However, these differences may arise from a combination of electronic effects, Pt coordination, cocatalyst morphology, or the reductive pretreatment used before CO‐DRIFTS measurements. Based on the combined structural, photophysical, and catalytic evidence, we nevertheless propose that the Cu core promotes the formation of an Ohmic‐like contact between Pt and SrTiO_3_, as illustrated in Scheme S1. Because the barrier height and interfacial charge distribution were not directly measured, this interpretation should be regarded as a plausible model rather than the definitive proof of an ideal Ohmic contact.

### 2.3 Catalyst Deactivation and Activation

Collectively, activity tests, TRPL, CO chemisorption, EDS, and electron microscopy consistently support a structure–activity relationship, wherein Cu‐core/Pt‐shell cocatalysts enhance charge separation and increase the photocatalytic activity. During long‐term photocatalytic testing, however, all catalysts showed fast initial deactivation during the first ~5 h TOS followed by either a rather stable performance (04Pt_01Cu_GD, 06Pt_02Cu_GD), slow deactivation (01Pt_PD), or even pronounced activation (02Pt_01Cu_GD/03Pt_01Cu_GD, peaking at 20–30 h) as shown by the MeOH conversion versus TOS profiles in Figures 1C and S6. In order to elucidate the activation/deactivation behavior and identify mechanisms to prevent deactivation, it is necessary to obtain information about the potential causes of this behavior. Therefore, in situ IR spectroscopy was performed in DRIFTS mode under reaction conditions (Table S5). Before illumination, the catalyst was exposed to a MeOH/H_2_O/He feed at 100 °C for 30 min. The corresponding spectra reveal the formation of adsorbed MeOH‐derived species (Figure S18). The negative band at 3700 cm^−1^ is assigned to ν(O─H), while the four positive bands around 2920, 2820,1150, and 1050 cm^−1^ are assigned to the asymmetric and symmetric vibrations, *ν*_as_(C─H) and *ν*_s_(C─H), and ν(C─O) of methoxy and physisorbed and gas‐phase MeOH [79, 80, 81, 82]. The rather sharp and stable bands at 3950–3500 and 1900–1300 cm^−1^ are originating from gas‐phase water [79, 83]. While the formation of the band at 2085 cm^−1^, assigned to CO adsorbed on Pt hints at the thermal decomposition of MeOH, bands related to gas‐phase CO or FA are absent. Additionally, a band assigned to CO_2_ is observed at 2350 cm^−1^, consistent with results obtained from the flat‐plate reactor, where the concentrations of FA, CO, and H_2_ are below the detection limits under dark conditions and only traces of CO_2_ are detected (Figure S19). This indicates that the contribution of the dark reaction to the measured gas‐phase product formation was negligible. The temporal evolution of the DRIFT spectra under reaction conditions for the 01Pt_PD and 04Pt_01Cu_GD samples is shown in Figure 5 (note that, as background, a spectrum obtained under inert conditions after the H_2_O cleaning procedure was taken). Utilizing a spectrum recorded after 30 min under reaction conditions in the dark as the background (Figure S20), however, reveals clear light‐induced changes with the negative bands at 2951 + 2848 and 2917 + 2817 cm^−1^ pointing toward the consumption of physisorbed MeOH and methoxy groups [79, 80, 81, 82]. Importantly, the background subtraction removes species already present at the surface in the dark, and therefore, the resulting difference spectra are interpreted as changes relative to the dark reaction state. As illustrated in Figure 5, the formation of formate (broad bands around 1550–1650 cm^−1^), which has been identified as a primary cause of long‐term deactivation [26], is evident over time for the 01Pt_PD sample. The difference spectra provided in Figure S20 also reveal formate formation for the 04Pt_01Cu_GD sample by the positive bands at 2864, 1622 + 1524, and 1390 + 1330 cm^−1^ that are assigned to ν(C─H), *ν*_as_(C─O), and *ν*_s_(C─O), respectively [79, 84, 85]. Consequently, the slow deactivation of the samples prepared by GD over prolonged TOS is likely also caused by formate accumulation. In light of the continuous growth of the formate bands, the rapid deactivation observed at the beginning, and the activation of some of the GD samples, it is evident that the presence of formate alone cannot account for these phenomena. Contrasting the appearance of the formate‐related vibrations after extended TOS, a positive band at 2900 cm^−1^ is already present before illumination. The band is assigned to CO on Pt, in agreement with the CO‐DRIFTS measurements discussed above. The appearance of the band suggests that thermal decomposition of MeOH over the various samples occurs already in the dark. Similarly, to the different shape of the band observed during CO‐DRIFTS, the band shape appears to strongly depend on the sample preparation procedure, as also discussed above. While the Pt‐CO band of the 01Pt_PD sample evolved in shape and shifted to lower wavenumbers during prolonged illumination, the Pt‐CO band of the 04Pt_01Cu_GD sample appeared to be fixed in position, although changes in the band shape were also observed. Given the strong correlation between the cocatalyst structure and photocatalytic activity revealed by activity testing and TRPL measurements of the different samples, the structural changes of the cocatalyst are tentatively assigned as the cause of deactivation and presumably photocatalyst activation. This activation likely occurs in parallel to the accumulation of formate, yet at a different time scale.

**FIGURE 5:**
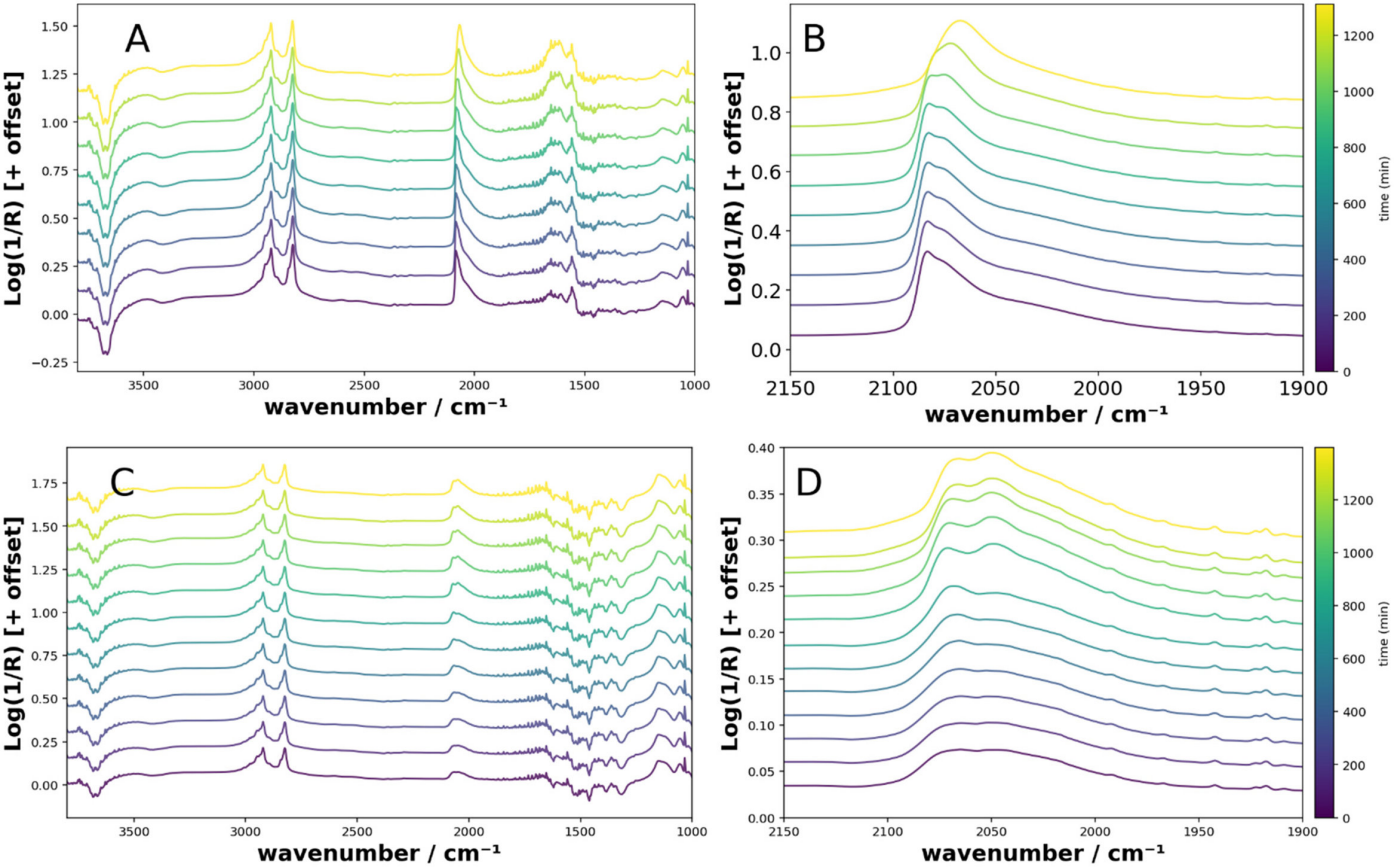
DRIFT spectra obtained for (A,B) 01Pt_PD and (C,D) 04Pt_01Cu_GD during photocatalytic MeOH dehydrogenation at 100 °C using a reactant feed containing 1.5% MeOH and 4.5% water in He at a flow rate of 10 mL/min. Note that the background spectra were taken in inert atmosphere after the H_2_O cleaning procedure.

CO evolution during the reaction (~30‐40 ppm in the flat plate reactor, Figure S21) enables Pt‐CO probing under low‐coverage, reaction‐relevant conditions, and thus, a detailed analysis of the changes in the Pt‐CO band was performed using the peak model described above (Table 2) for the samples 01Pt_PD and 04Pt_01Cu_GD. Examples of the deconvoluted Pt‐CO signals determined after different TOS are shown in Figure 6B,C,E,F. Generally, the total area of the Pt‐CO band scales with Pt loading (01Pt_PD > 04Pt_01Cu_GD; Figure S22). For the 01Pt_PD sample, peak deconvolution (Figure 6A) suggests that during the initial ~400 min, Pt_HC_ increases (peak 4, 2085 cm^−1^) as the LC contribution (peak 3, 2070 cm^−1^) decreases. The change in Pt distribution is strongly correlated with the conversion, which dropped sharply within the initial ~400 min TOS (Figure 6A). CO on HC Pt and bridged CO (peak 5 and 1) show a decline in intensity. This overall intensity decline suggests sintering, presumably resulting in a lower Pt dispersion. While HC Pt (100) (increase of Peak 4) seems to be formed from HC Pt (111) (decrease of Peak 5) and LC Pt from edges (decrease of Peak 3), at a later stage, Pt edges (Peak 3) likely form from HC Pt (100) (Peak 4). For the 04Pt_01Cu_GD sample (Figure 6D), the peak 3 and 4 increased sharply upon illumination and maximized in intensity after ~200 min TOS. Importantly, the time scale of deactivation strongly correlates with the increase in the intensity of both peaks (Figure 6D). Subsequently, only a minor decrease of peak 3 and 4 with TOS is observed, which correlates with a sustained stable conversion of MeOH, which is presumably also hinting at an inhibiting effect of HC Pt sites (Peak 4). The results of the analysis of the 01Pt_PD sample suggested a positive effect of LC Pt from edges (Peak 3) on the MeOH conversion, but a decrease in conversion alongside an increase in the intensity of Peak 3 observed for 04Pt_01Cu_GD contradicts this correlation. In fact, for the 04Pt_01Cu_GD sample, TEM measurements performed before and after 48h TOS (Figure S23A–D) verified restructuring of the core–shell catalysts. The hexagonal Pt NPs observed before the reaction disappeared, and instead, smooth Cu‐core/Pt shell structures were observed post‐reaction. In addition, small residual Pt NPs (Figure S24) were revealed. EDS analysis confirmed a sustained Pt/Cu ratio and highlighted an increase in the carbon content, indicating that CO adsorption is stabilized after Pt restructuring (Figure S23E).

**FIGURE 6:**
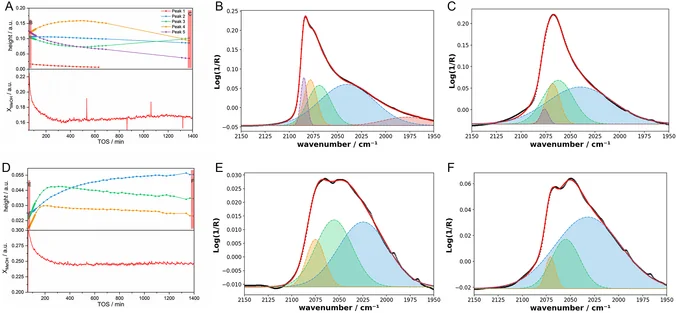
Temporal course of peak heights obtained from deconvolution of the Pt‐CO band during in situ methanol dehydrogenation and MeOH conversion in the flat plate reactor (1.5% MeOH/4.5% H_2_O, 100 °C, 365 nm) for (A) 01Pt_PD and (D) 04Pt_01Cu_GD. Temporal peak heights reveal site‐specific dynamics correlating with conversion shown in the TOS profiles (Figure S19). The deconvoluted Pt‐CO band obtained after 30 min in reaction atmosphere and after 1200 minutes under illumination as highlighted in (A) and (B). (B,C) for the 01Pt_PD and (E,F) for the 04Pt_01Cu_GD sample, respectively.

In situ probing and subsequent deconvolution of the Pt‐CO band reveal dynamic changes in the Pt surface during operation. These changes are evident from the temporal evolution of the individual peak contributions (Figure 6) and the additional peak characteristics summarized in Figures S25 and S26. CO‐induced restructuring of Pt nanoparticles is a known phenomenon [57]. In particular, the increasing fraction of low‐coordination Pt sites during CO exposure is consistent with the increase in peak 2 assigned to low‐coordination Pt sites in the GD samples. The initial increase in the high‐coordination Pt contribution associated with peak 4 may indicate a transformation from a less crystalline toward a more crystalline particle structure. The Pt(100) facet is known to undergo reconstruction even at temperatures below 100 °C [86, 87]. CO‐induced Pt restructuring has been reported to be reversible, with the reconstructed structure being thermodynamically stabilized primarily in a CO‐containing atmosphere [57]. In the present system, however, the persistence of strongly bound carbon‐containing species may hinder the complete recovery of the initial Pt surface. Post‐reaction EDS revealed an increase in the carbon signal, but this observation does not distinguish between coke‐like deposits, strongly adsorbed CO‐derived species, formate‐related species, or residual organic material. While Raman spectroscopy is generally suitable to identify graphitic or coke‐like carbon, it is noted that reliable Raman analysis is challenging because of the low catalyst loading and strong fluorescence from the glass substrate, obscuring the weak catalyst signal. We therefore do not assign the post‐reaction carbon enrichment specifically to coke deposition. Nevertheless, the combined temporal correlation between the Pt‐CO changes, carbon enrichment, and activity loss suggests that strongly bound carbon‐containing species may contribute to the persistence of the reconstructed Pt surface. Continuous removal or suppression of CO adsorption at Pt sites and/or deposition of other carbon‐containing species may therefore help preserve the catalytically favorable Pt surface and reduce long‐term deactivation [87]. On the basis of the present data, avoiding CO‐associated deactivation could potentially recover approximately 10%–30% of the initial activity, as estimated from the observed decrease in AQY for H_2_ and FA.

In summary, the present in situ DRIFT study has demonstrated that CO‐induced Pt reconstruction is a plausible cause of photocatalytic deactivation. To the best of our knowledge, this phenomenon has not been previously reported in the context of photocatalysis. However, it is likely to impact photocatalysis beyond MeOH hydrogenation. Furthermore, the observed restructuring provides valuable insights and opens up new avenues for the optimization of Pt‐based photocatalysts. For instance, future studies should concentrate on enhancing the light‐harvesting properties by preventing CO‐driven Pt restructuring, in addition to employing advanced SrTiO_3_, such as Al‐ or Mg‐doped SrTiO_3_, which is recognized for its exceptional activity in photocatalytic water splitting [88, 89, 90]. For the former, it is crucial to impede CO adsorption on Pt while facilitating proton reduction and H_2_ desorption. Strategies that presumably mitigate CO adsorption might include encapsulation of Pt using inorganic permeable oxide shells or pulsed illumination utilizing 440 nm illumination to accelerate CO deposition [88, 91, 92, 93, 94, 95]. In light of the available literature and the findings of this study, it is evident that photocatalytic FA production is a reaction of significant promise, with the potential to mitigate the environmental impact of FA manufacturing.

## 3 Conclusion

This study introduces Cu‐core/Pt‐shell cocatalysts on SrTiO_3_ as an efficient cocatalyst enhancing charge separation and extending carrier lifetimes, presumably by fostering the formation of Ohmic‐like metal‐semiconductor contacts. We show that by optimizing the Pt:Cu ratios, the anaerobic, thermally assisted photocatalytic MeOH dehydrogenation activity to the value‐added selective oxidation product formaldehyde increases by 50% compared to conventional photocatalysts utilizing photodeposition for Pt immobilization (24% vs. 16% conversion and 65% formaldehyde selectivity). Advanced characterization confirm the successful formation of a Cu‐core/Pt‐shell morphology and a distinct site‐specific Pt dispersion. Importantly, DRIFTS and postmortem TEM reveal CO‐induced Pt reconstruction, which is proposed to be a major deactivation pathway. Overall, this work extends Ohmic‐contact design to new semiconductor/metal/reaction combinations and emphasizes the importance of cocatalyst engineering and CO resilience for a stable, gas‐phase photocatalytic conversion of MeOH.

## 4 Experimental

### 4.1 Synthesis

Commercial SrTiO_3_ is used in this study as a light absorber. To deposit Cu on the SrTiO_3_ surface, a previously reported in situ photodeposition approach was applied [96]. Briefly, 1.638 g of SrTiO_3_ was suspended in 410 mL of water (HPLC grade) and 40 mL of MeOH, and the required amount of Cu(NO_3_)_2_ solution (5 mg Cu/mL, Thermo Scientific, 99%) was added. The suspension was sonicated for 1 h before air was removed from the system by purging with pure N_2_ (50 mL/min) for 30 min. The suspension was illuminated for 60 min under a constant N_2_ flow using a 500 W Hg lamp (59 mW cm^−2^, Consulting Peschl). The formation of metallic copper was indicated by a brownish coloration of the suspension. Cu‐core/Pt‐shell structures were prepared by a galvanic displacement approach. After the deposition of Cu, 2.6 mL of glacial acetic acid (99.7%, Fischer Chemicals) was added in the dark to facilitate the removal of Cu oxides. The suspension was stirred for 10 min before the required amount of H_2_PtCl_6_ solution (14 mg Pt/mL, Sigma Aldrich 99.995%) was added to the suspension under a N_2_ flow. This resulted in a fast color change from brownish to off white, indicating the oxidation of metallic Cu. The suspension was stirred for another 30 min before recovering the photocatalyst by vacuum filtration, washing with 1 L of water (HPLC grade), and drying under vacuum at 50 °C. The samples are labeled with respect to the quantity of cocatalyst and the synthesis approach.

### 4.2 Characterization

Transmission electron microscopy (TEM), scanning transmission electron microscopy in high‐angle annular dark‐field mode (STEM‐HAADF), secondary electron imaging (SEI), and energy‐dispersive X‐ray spectroscopy (EDS) were conducted using a JEOL JEM‐2800 equipped with a Schottky field emission gun operated at 200 kV. Conventional TEM images were recorded with a Gatan OneView IS camera. STEM‐HAADF images were acquired using a high‐angle annular dark‐field detector at collection angles of 70–200 mrad, and elemental mapping was performed with dual silicon drift detectors.

X‐ray photoelectron spectra (XPS) were recorded using a HiPP‐Lab NAP‐XPS system equipped with a monochromatic Al Kα X‐ray source (1486.6 eV). The sample was embedded in a standard gold grid (300 mesh, 3.05 mm diameter) commonly used for TEM measurements by filling the grid and applying gentle pressure. Survey scans were acquired with a pass energy of 500 eV, and the region spectra were collected at 200 eV. Spectral acquisition was performed over a circular area of 0.3 mm in diameter at a pressure of 3 × 10^−9^ mbar.

Diffuse reflectance UV–vis spectra were measured in the range of 800–200 nm with a data interval of 1 nm and an integration time of 0.32 s per step. A Perkin Elmer Lambda 650 spectrometer with a praying mantis geometry was used, operated by the UV WinLab Software. As a 100% reflectance standard, BaSO_4_ (Sigma Aldrich, 97.5%) was used.

TRPL experiments were performed using a 266 nm excitation source and a streak camera setup (Hamamatsu, C10910) integrated into a home‐built setup. Further details of the TRPL measurements can be found in the Supporting Information.

DRIFTS measurements were performed in a setup described in detail elsewhere [26]. Briefly, a Nicolet iS50 equipped with a LN2‐cooled MCT detector and a Harrick high‐temperature reaction chamber in the praying mentis mirror geometry were used. For CO‐DRIFTS measurements, the samples were treated in synthetic air at 200 °C for 1 h, followed by treatment in H_2_ at 200 °C for 1 h to remove residual organic species from the surface. Before CO adsorption, a background was recorded at 35 °C. CO (1000 ppm in N_2_) was adsorbed for 15 min, followed by purging with N_2_ for 40 min. Deconvolution was performed by a custom python code. In situ MeOH dehydrogenation measurements were performed in the same setup using 1.5 % MeOH and 4.5 % water in 10 mL min^−1^ He as feed gas. The desired feed composition was obtained using a heated saturator system. The saturator was filled with 40 mL of water and 3.340 mL of MeOH and heated to 33.2 °C (temperature was calculated according to the Antoine equation to yield the desired concertation) using a water bath controlled by a thermostat (Alpha RA 8, Lauda). Before the measurements, the photocatalyst was pretreated using water only. The different steps of the in situ DRIFTS measurements are also summarized in Table S4.

CO chemisorption was performed in the pulsed mode using 1% CO in N_2_ at RT after H_2_ TPR up to 350 °C. 20 pulses were recorded, where the last 5 pulses were used as calibration, assuming a saturated surface was achieved after 15 pulses. The bulk composition was determined by inductively coupled plasma mass spectrometry (ICP‐MS) analysis using a PerkinElmer NexION 350X after dissolving the samples in hydrofluoric acid.

X‐ray total scattering experiments were performed at the P21.1 beamline of the storage ring PETRA III (DESY, Germany). A (1 × 1) mm^2^ beam was used at an energy of 101.5 keV. A PerkinElmer XRD1621 amorphous Si flat panel X‐ray detector was used at a distance of 406.0 mm from the sample. The samples were packed in 0.8 mm diameter kapton capillaries, and an empty one was taken as background. The sample‐to‐detector distance was calibrated using a Ni standard. The detector distance was refined and the data was azimuthally integrated into 1D patterns using pyFAI [97, 98]. The total scattering data were Fourier transformed using xPDFSuite [99] to obtain PDFs over a Q‐range of 0.96 (*Q*_min_) to 20 Å^−1^ (*Q*_max_) with a *Q*_max_ Inst of 24 Å^−1^, and rPoly of 1.9. The instrumental parameters were determined by fitting against a Ni standard and refined to a *Q*_broad_ of 0.0045 and *Q*_damp_ of 0.0362. Total scattering data of the untreated SrTiO_3_ were measured under identical conditions as 04Pt_01Cu_GD, containing ~0.1 % of the nanoparticles, and used as background to generate the d‐PDF data. PDFgui [100] was used to refine the d‐PDF data and to simulate the PDFs of fcc Pt [101], CuPt_7_ [102], Cu_3_Pt [103, 104], and Cu [105] using the experimentally determined *Q*_broad_ and *Q*_damp_.

### 4.3 Photocatalytic Performance Measurements

The photocatalyst was immobilized by spray coating a catalyst isopropanol suspension onto a sand‐blasted quartz glass plate using an ultrasonic spray coater (USI PRISM 400), resulting in a loading of 60–80 mg (1–1.3 mg cm^−2^). The coating parameters are detailed in Table S1. The immobilized photocatalysts were tested using a flow reactor equipped with a quartz window for illumination. As light source, a custom‐made LED array was used (30 LEDs, 365 nm, 52.93 mW cm^−2^). The supply of water and MeOH in the N_2_ stream was achieved by temperature‐controlled saturators. A detailed description of the setup used for MeOH dehydrogenation can be found elsewhere [25]. Before MeOH dehydrogenation, the immobilized photocatalysts were treated in 4.5 % water vapor at 100 °C while illuminating for 18 h to remove organic residues from the photocatalyst surface. The reaction conditions were chosen in agreement with the recent work of Deitermann et al. [25], suggesting that a good agreement between selectivity and conversion as well as durability, is achieved at a reaction temperature of 100 °C. The feed gas composition was adjusted to *y*_MeOH_ = 1.5 % and *y*_H2O_ = 4.5 %, using a gas flow rate of 50 ml/min Note that water is added to the feed gas to ensure proton transport on the photocatalyst surface, which was found to be crucial in earlier work [25]. The photocatalysts were tested for at least 48 h. The exhaust stream was analyzed by an FTIR spectrometer (Thermo Scientific, Nicolet 6700) to quantify carbon‐based species and a multichannel gas analyzer (Emmerson, X‐Stream) for the quantification of hydrogen.

## Funding

This study was supported by the Ministerium für Innovation, Wissenschaft, und Forschung des Landes Nordrhein‐Westfalen.

## Conflicts of Interest

The authors declare no conflicts of interest.

## Supporting information

Supplementary Material

## Data Availability

The data that support the findings of this study are available from the corresponding author upon reasonable request.
