# Supplementary material for: Decorating SrTiO3 With Cu@Pt Core‐Shell Cocatalysts to Promote the Selective Thermally Assisted Photocatalytic Methanol Dehydrogenation to Formaldehyde
Source: ChemSusChem. 2026 Sep 30;19(19):e71077. doi: 10.1002/cssc.71077 (PMC13627283; doi:10.1002/cssc.71077)
Supplement: Supplementary file 1 [file CSSC-19-e71077-s001.pdf]

Supporting Information

Decorating $SrTiO_3$ with Cu@Pt core-shell cocatalysts to promote the selective thermally assisted photocatalytic methanol dehydrogenation to formaldehyde

Yannik Haver[a], Zhangjie Zhai[a], Robert Rinio[a], Jorik Schaap[b], Fernando I. Saldaña[c], Andrea Kirsch[d], Kasper Wenderich[b], Bastian Timo Mei*[a]

Experimental Details

Time resolved photoluminescence has been done using a setup described in detail elsewhere.[1] The TRPL experiments were performed using a 266 nm excitation source. The 266 nm light was generated through second-harmonic generation of a 532 nm laser. The output of a Fianium laser (FP-532-s, center wavelength 532 nm, pulse duration of 30 fs, 80.3 Mz repetition rate) was focused through a lens on a 5mm thick KDP crystal (Edmund Optics, 12 x 12 x 5mm, 532nm SHG, Type I KDP Nonlinear Crystal). The obtained UV light at 266 nm was sent via three mirrors (Thorlabs, MBI-K04) and a filter (Thorlabs, FGUV11-UV) to remove any residual light from the 532 nm laser. The light was then focused through a 50 mm focal distance quartz lens on the sample, which was contained in a quartz cuvette. Photoluminescence was collected using two 2 inch diameter 50 mm focal length glass lenses and focused on a spectrograph (Acton SP2300, Princeton Instruments, slit width set at 100 μm) using a grating with 50 lines/mm blazed at 600 nm. The output of the spectrograph was sent into a streak camera setup (Hamamatsu, C10910).

Before measurements, we confirmed there was no background signal from the quartz cuvette itself in our measurements. Prior to measurements the calibration of the spectrograph was checked with a Hg/Ar calibration lamp (Oriel, LSP035).

For the data collection, the nano powders were loaded into the quartz cuvette and measured as is. All experiments were performed on multiple spots on the sample. Power-dependent measurements were performed to verify if the spectrum is intensity-dependent , however no such trends were found. During measurements we used 500 mW of 532 nm light, yielding approximately 100 μW of 266 nm light on the sample. All experiments were performed on the same day. The time window in the experiment was set at 2 ns and data points are collected every 4 ps. The slid width before the photocathode of the streak camera was set to 200 μm yielding a time resolution of 30 ± 1 ps. Data analysis was performed using the open source python package Pyglotaran.[2] We analyzed the spectra using global analysis, wherein we used a three component parallel decay model to fit the data.

Table S1: Parameters for ultrasonic spray coating.

Suspension loading	1.5 mg mL^{-1}
Liquid flow	1.5 mL s^{-1}
Head speed	75 mm s^{-1}
Head height	20 mm
Air pressure	12 psi
Coated area	60 x 100 mm^2
Volume sprayed	175 mL

Table S2: Standard reaction conditions used for catalytic performance test in the gas phase.

**Reaction condition**	**Value**
Reactor temperature T / °C	100
UV light intensity I / mw cm^{-2}	52.9
Total volumetric gas flow $\dot{V}_{total}$ / mL min^{-1}	50
Methanol mole fraction y_{MeOH} / %	1.5
Water mole fraction y_{H2O}	4.5

Table S3: Comparison of AQYs for H2 and FA at the start of illumination and after 48h for selected samples with relative decrease.

**Sample**	**AQY / %**				**Deactivation**	
	initial		After 48h			
	H_2	FA	H_2	FA	H_2	FA
04Pt_01Cu_GD	2.72	1.68	2.35	1.47	13.60%	12.50%
02Pt_01Cu_GD	2.06	1.36	1.82	1.2	11.65%	11.76%
01Pt_PD	2.16	1.45	1.5	1.14	30.56%	21.38%

Table S4: Position of deconvoluted peaks after 15 min 1000 ppm CO/N_2 exposure (35 °C, blank-subtracted)

**Sample**	**Position / cm^{-1}**					
	Peak 1	Peak 2	Peak 3	Peak 4	Peak 5	Peak 6
01Pt_PD	1976.5	2045.0	2071.4	2084.2	2092.5	
02Pt_01Cu_GD	1992.0	2043.6	2066.9	2083.6	2094.0	2120.2
04Pt_01Cu_GD	1992.0	2045.8	2071.0	2080.4	2089.9	2120.8

Table S5: Overview of the conditions at the different steps during the in-situ MeOH dehydrogenation experiments.

**Step**	**Procedure**	**T / °C**	**Atmosphere**	**Duration**	**Saturator Components**	**Saturator T / °C**	**UV LED**
1	He Purge	35	He	30 min			off
2	Heat up	100	He	120 min			off
3	H_2O purge	100	H_2O/He	30 min	H_2O	32	off
4	H_2O clean	100	H_2O/He	18 h	H_2O	32	on
5	LED off	100	H_2O/He	30 min	H_2O	32	off
6	He Purge	100	He	10 h			off
7	MeOH/H_2O Purge	100	MeOH/H_2O/He	30 min	H_2O/MeOH	32	off
8	MeOH dehyd.	100	MeOH/H_2O/He	24 h	H_2O/MeOH	32	on
9	LED off	100	MeOH/H_2O/He	30 min	H_2O/MeOH	32	off

10	He Purge	100	He	10 h	off

Table S6: Ti and Sr amount determined by ICP-MS for selected samples and catalyst loading.

Sample	Sr / wt.-%	Ti / wt.-%	Catalyst loading / mg/cm^2
01Pt_PD	38.1	22.1	1.23
02Pt_01Cu_GD	40.4	23.7	1.00
03Pt_01Cu_GD	37.1	20.8	1.22
04Pt_01Cu_GD	40.8	23.9	1.48

The theoretical Sr/Ti mass ratio for stoichiometric $SrTiO_3$ is 1.83. The measured values of 1.70-1.78 are slightly lower than this theoretical value. According to the trace-metal analysis provided by the manufacturer, the commercial $SrTiO_3$ contains approximately 11,316 ppm trace metals, primarily Ca and Ba. Correcting the Sr/Ti mass ratio for the contribution of these heavier alkaline-earth elements gives an estimated value of approximately 1.79. This value is close to the theoretical ratio, and the remaining difference is within the uncertainty associated with ICP-MS analysis, sample digestion, and compositional heterogeneity. The measured Sr/Ti values therefore do not provide evidence for substantial $SrTiO_3$ non-stoichiometry. Since all photocatalysts were prepared from the same $SrTiO_3$ batch and compared under identical reaction conditions, surface acidity/basicity and oxygen-vacancy concentration that are closely linked to the stoichiometry were not independently quantified in this work to focus on the effect of Cu@Pt cocatalyst structure and interfacial charge separation. A detailed investigation of support acidity/basicity and oxygen-vacancy concentration will be considered in future work.

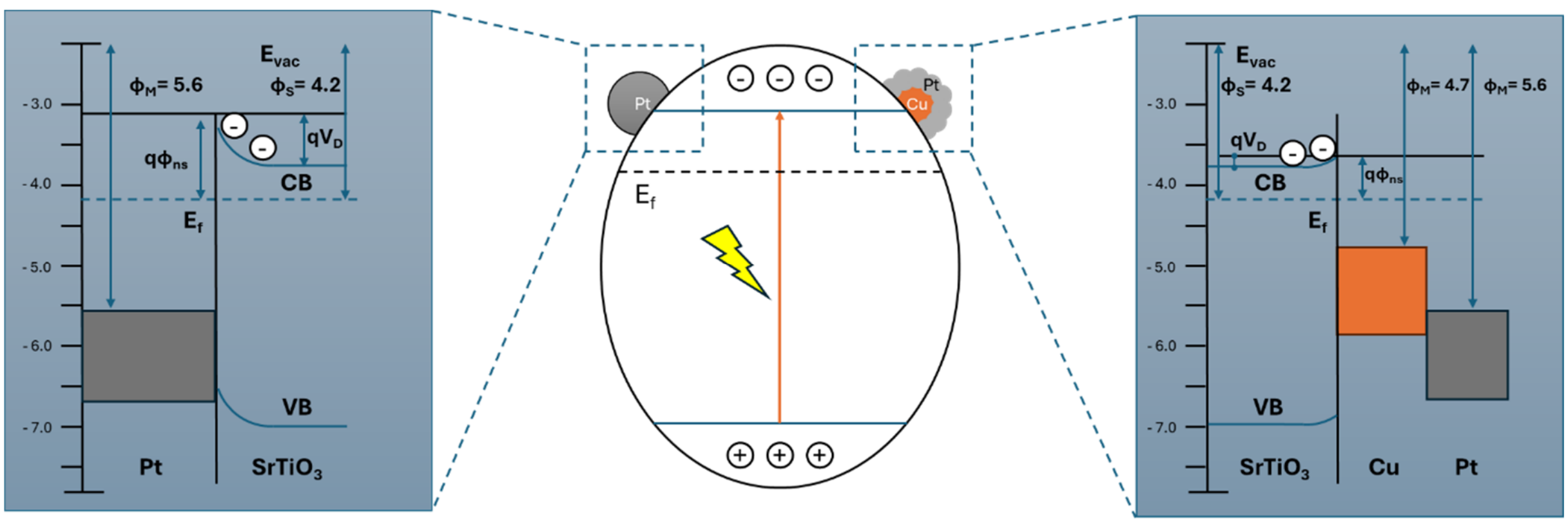

Scheme S1: Band bending schematics for Cu-core Pt-shell and just Pt NP cocatalysts. E_{vac}, E_f and ϕ denote the vacuum level, Fermi level, and work function, respectively (in eV).

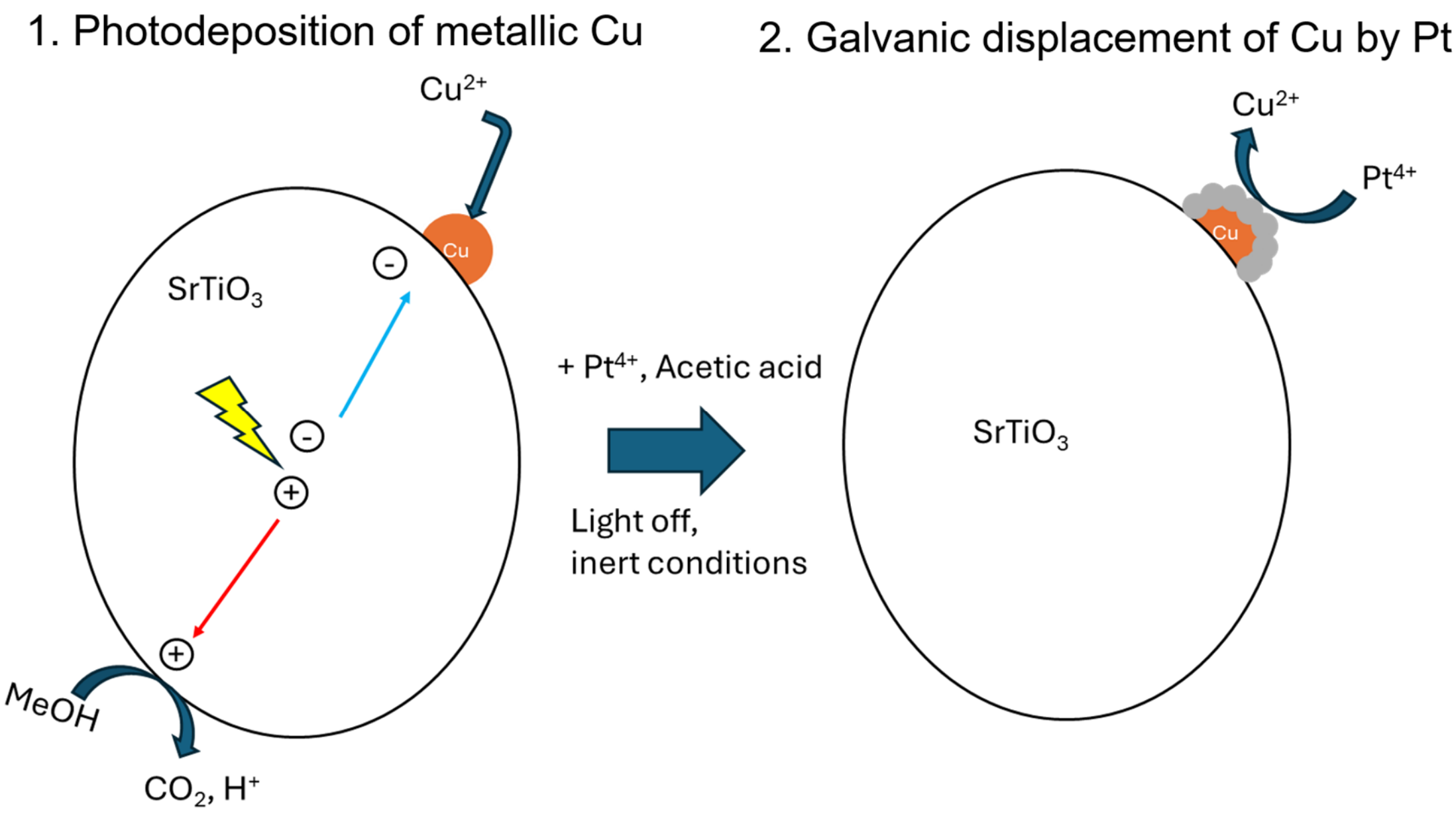

Scheme S2: Synthesis of Pt@Cu/$SrTiO_3$ via (i) Cu photodeposition on $SrTiO_3$ and (ii) Pt galvanic displacement.

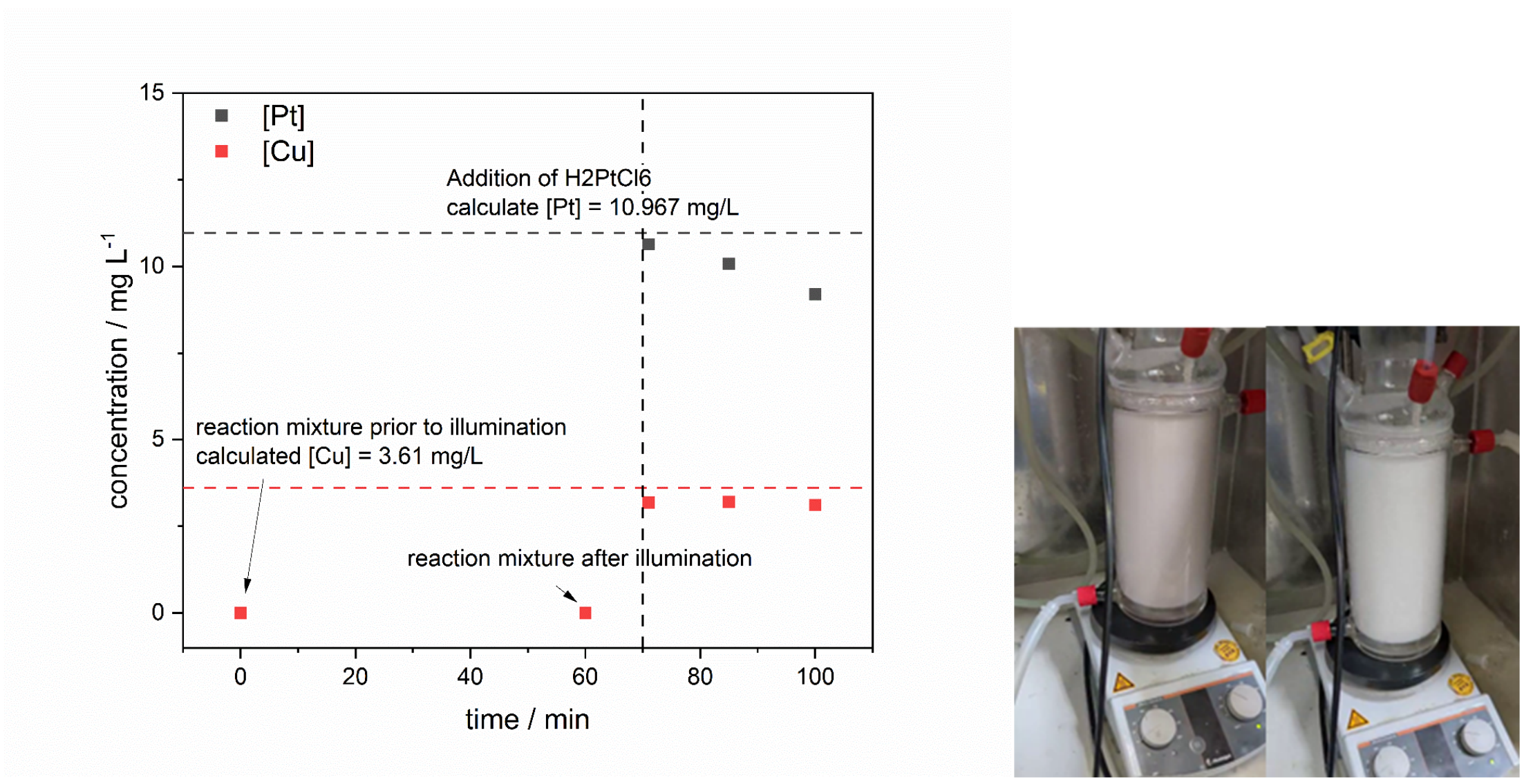

Figure S1: Cu and Pt concentrations in solution during synthesis of 03Pt_01Cu_GD. Inset: Color changes (white → pale brown → off-white).

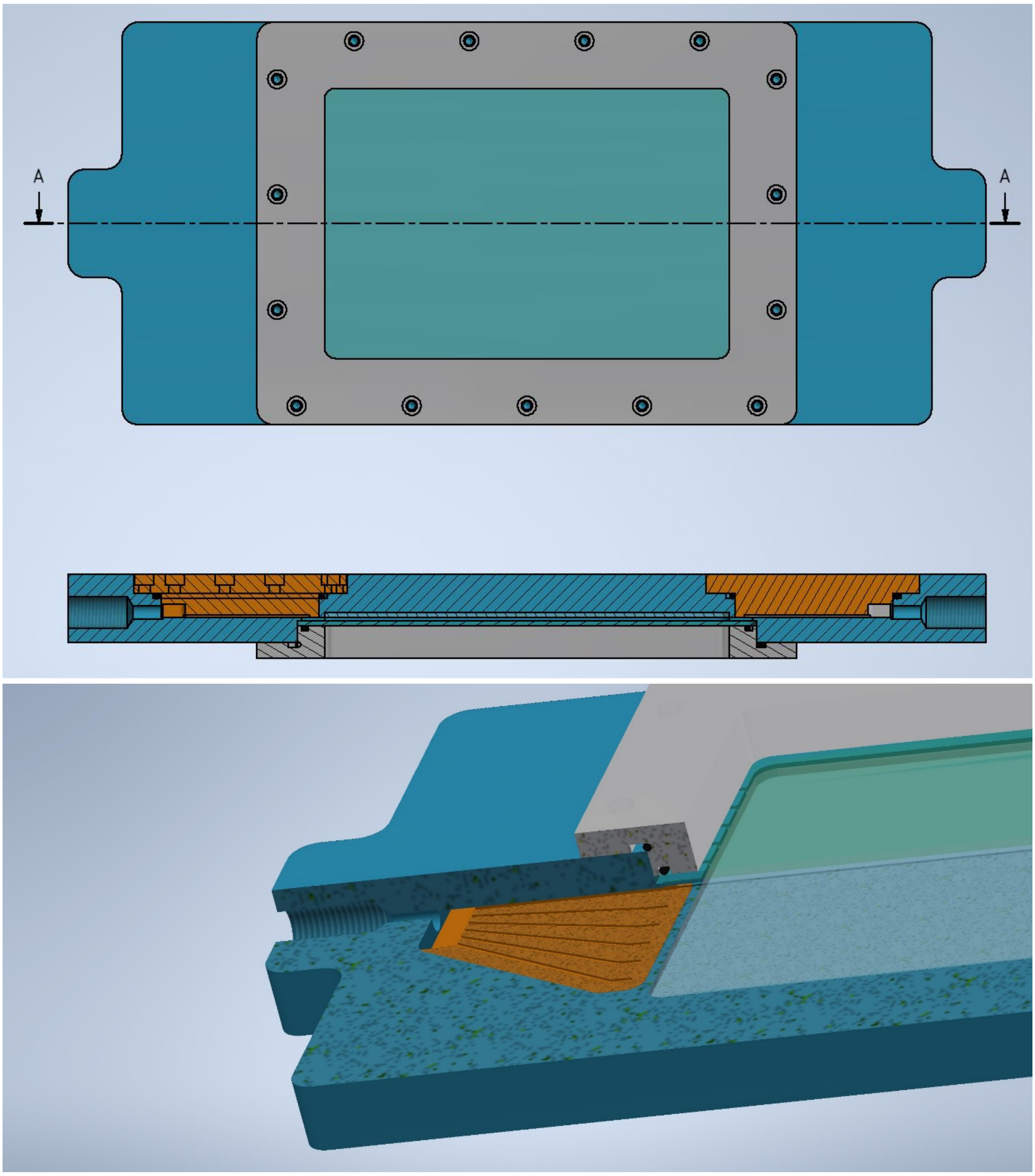

Figure S2: Technical drawing of the flat-plate reactor made of aluminum showing the gas flow channels (left) and a top view and a side view of the reactor showing the quartz window (right).

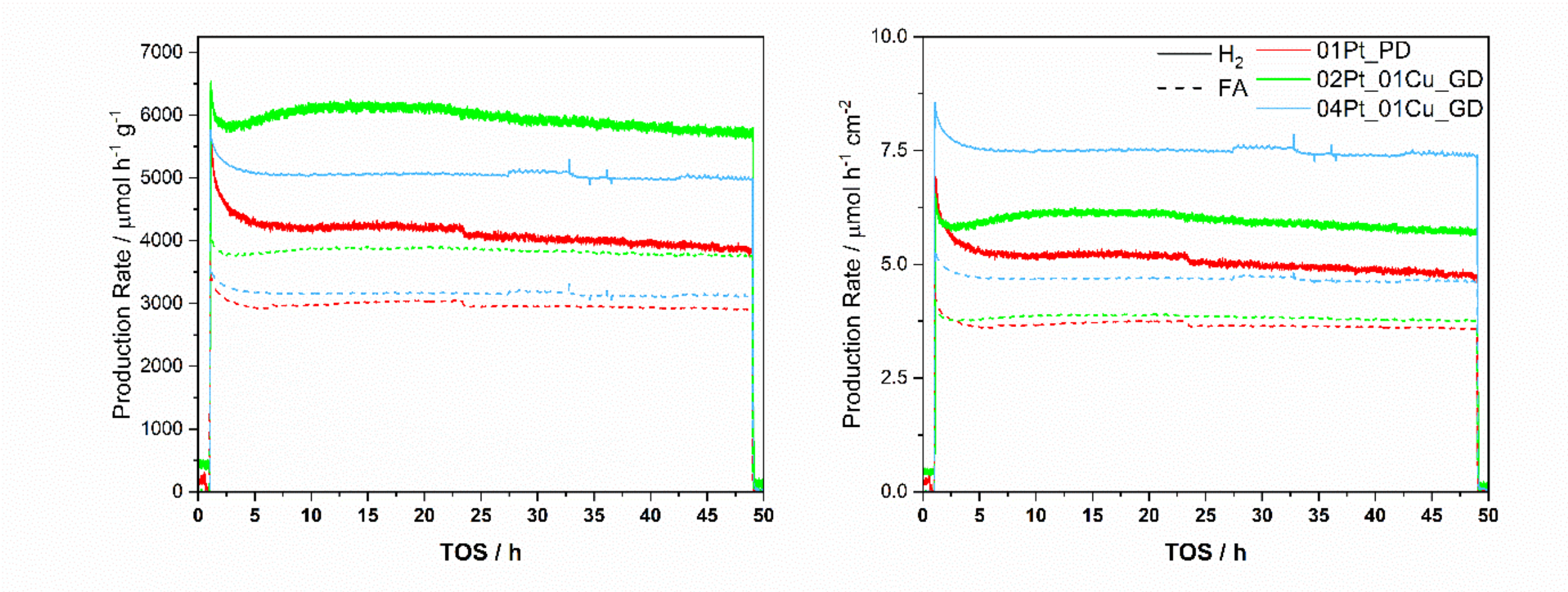

Figure S3: H_2 and FA production rates normalized to mass and area during methanol dehydrogenation over 01Pt_PD, 02Pt_01Cu_GD, and 04Pt_01Cu_GD.

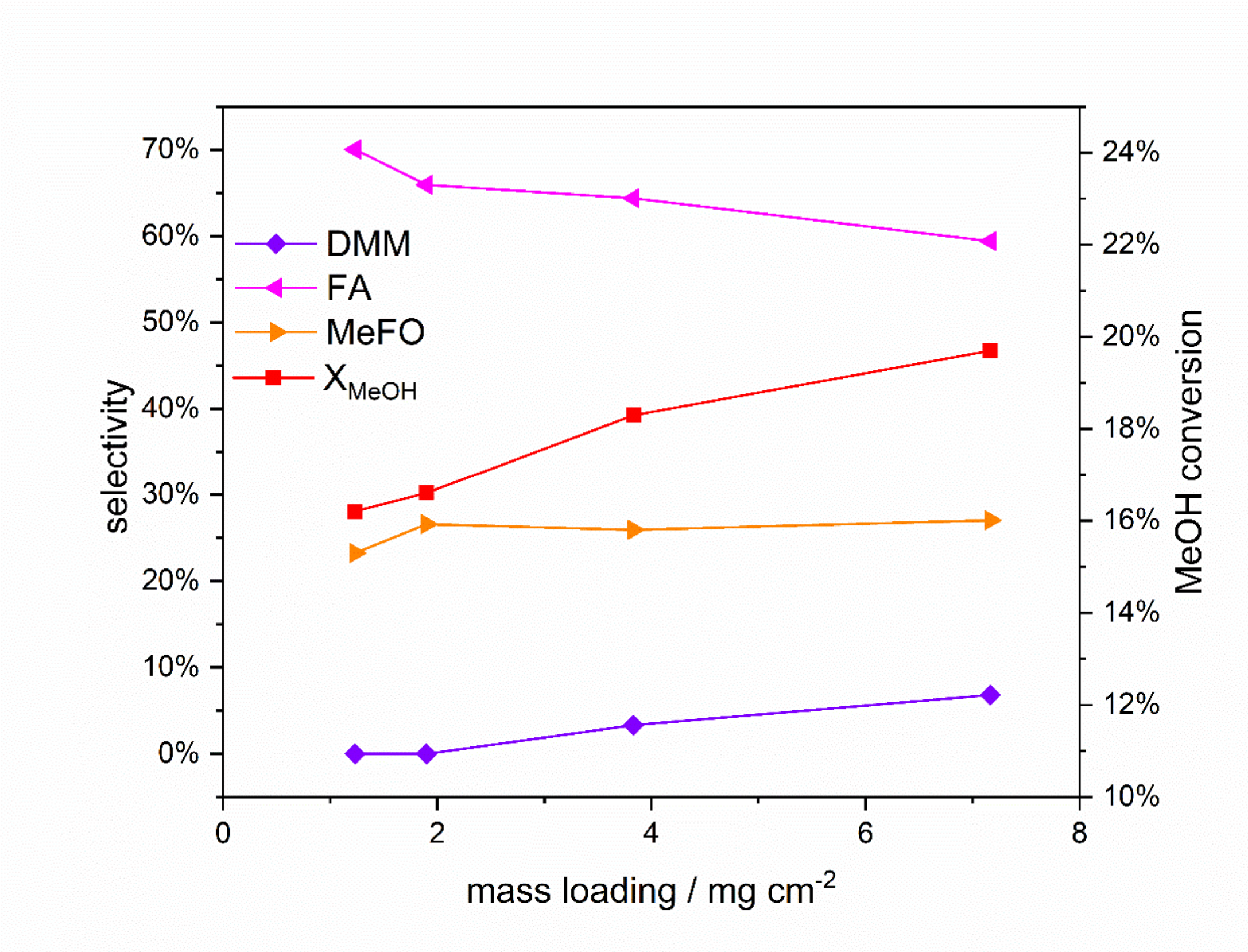

Figure S4: Selectivity and MeOH conversion after 48 h TOS during photocatalytic methanol dehydrogenation for different mass loadings of 01Pt_PD photocatalyst.

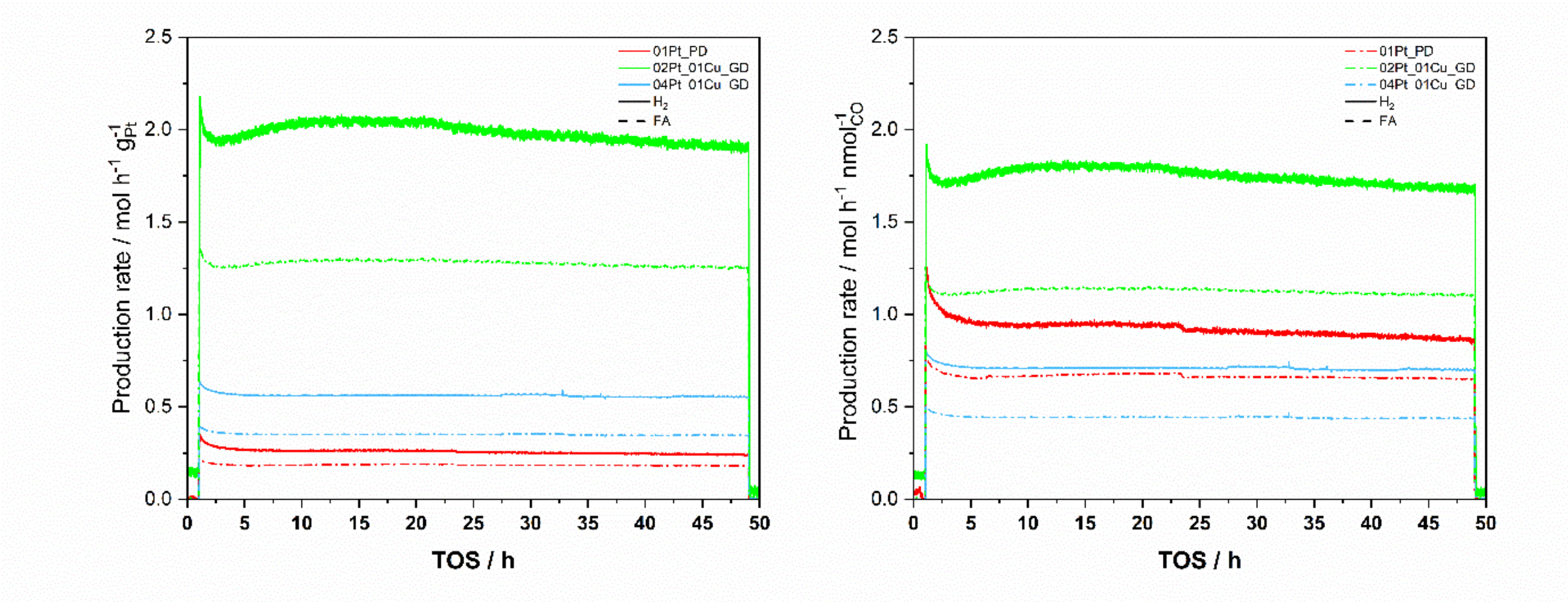

Figure S5: H2 and FA production rates normalized to the amount of Pt and Pt area (determined by CO Chemisorption) during methanol dehydrogenation over 01Pt_PD, 02Pt_01Cu_GD, and 04Pt_01Cu_GD.

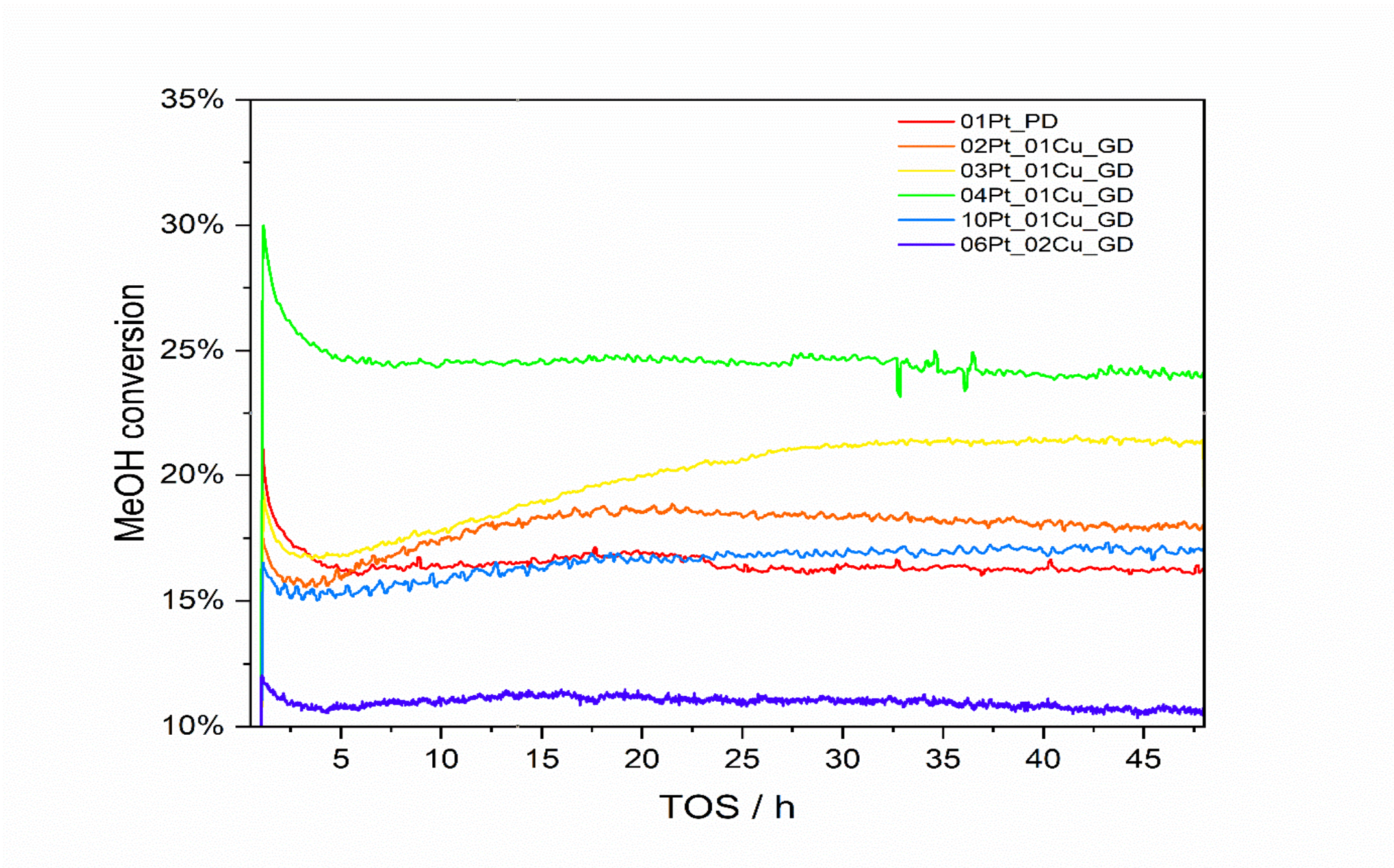

Figure S6: Methanol conversion vs. TOS for all tested samples during photocatalytic methanol dehydrogenation at 100°C.

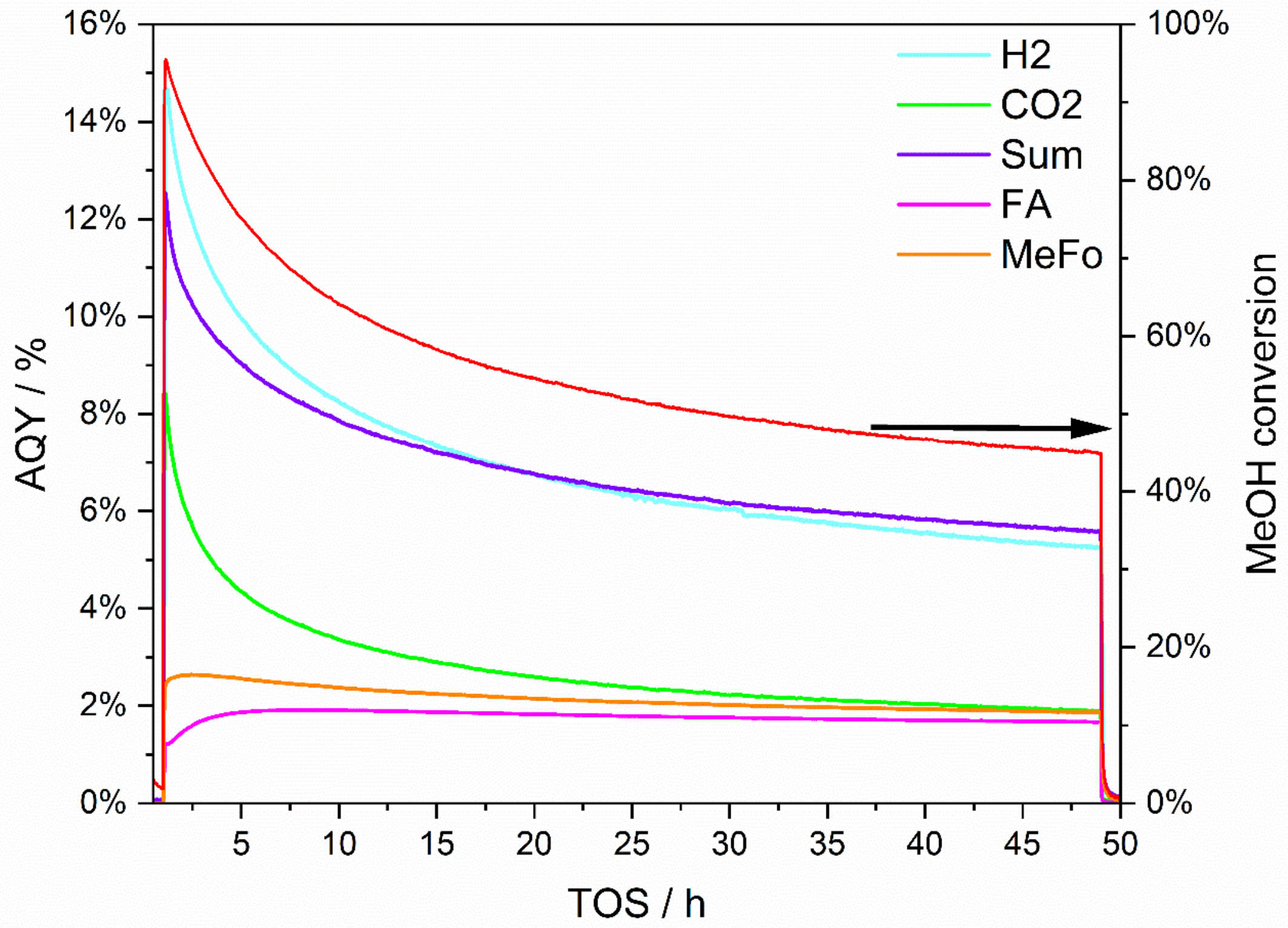

Figure S7: Apparent Quantum yield for H2, FA, CO2, MeFo, and MeOH conversion during thermally assisted anaerobic photocatalytic methanol dehydrogenation in the gas-phase at T = 100 °C over Pt loaded (0.1 wt.%) TiO_2 (P25) immobilized by doctor blading (6.67 mg cm^{-2}).

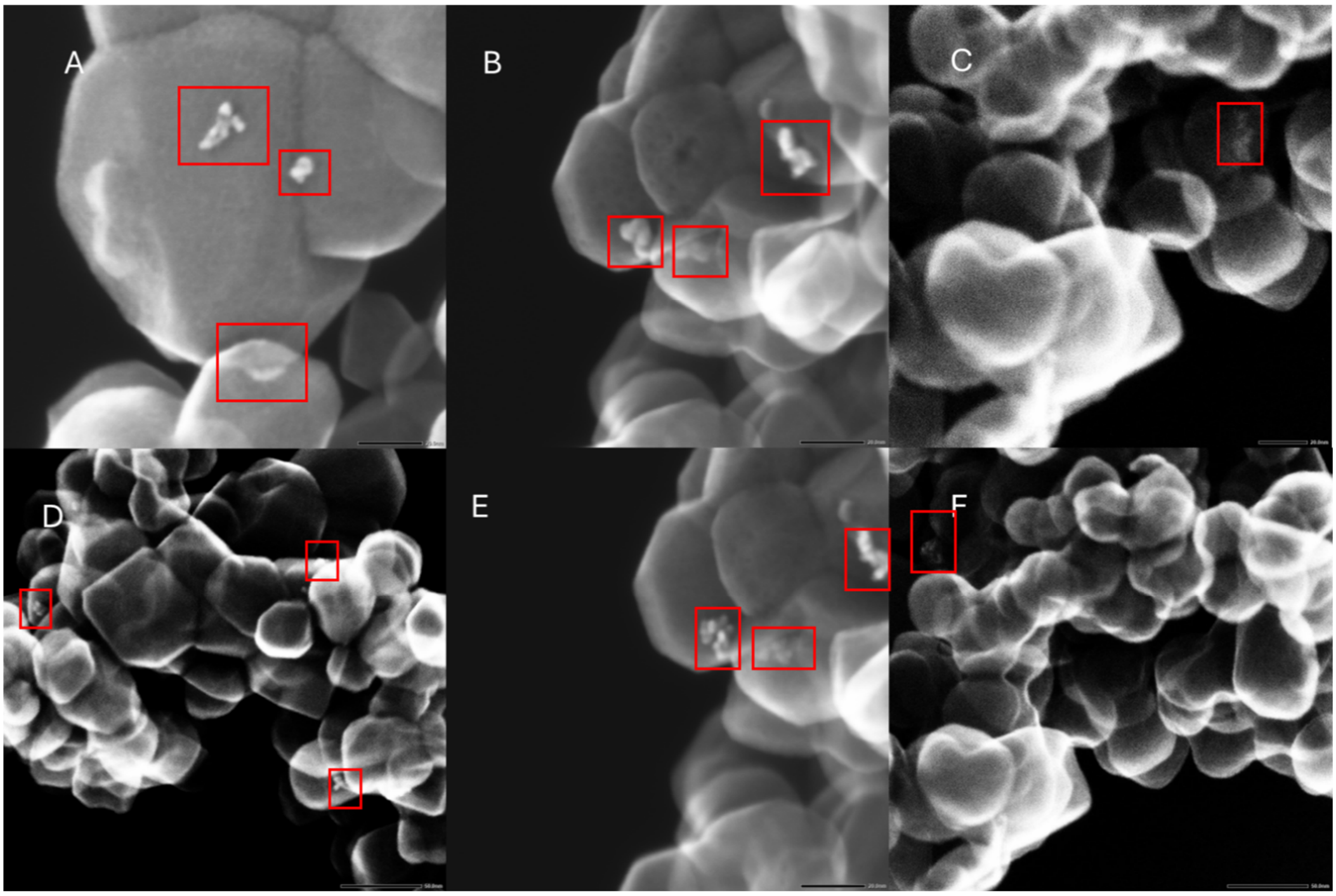

Figure S8: SEM images of the samples 03Pt_01Cu_GD (A,D), 04Pt_01Cu_GD (B,E), and 06Pt_01Cu_GD (C,E) .

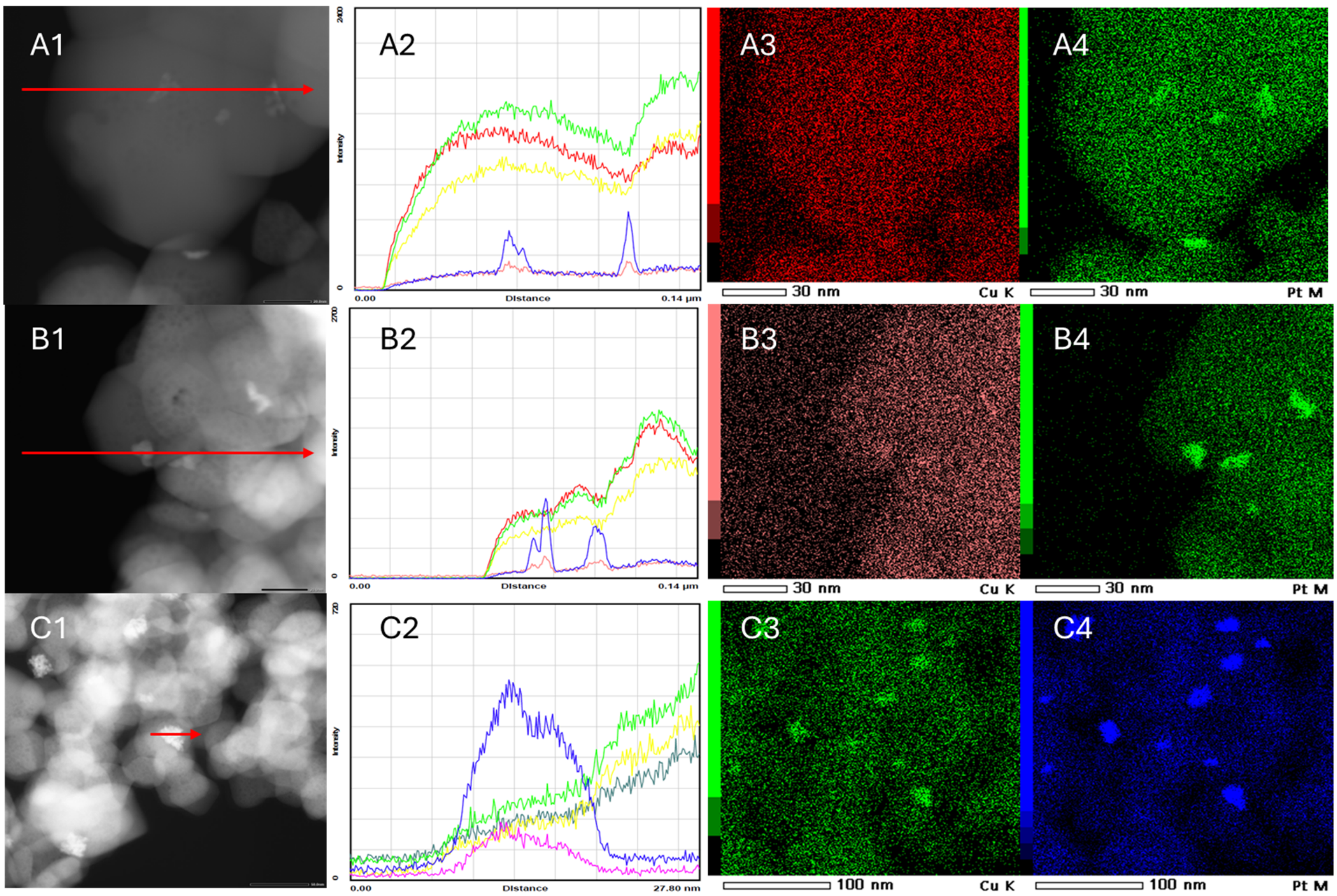

Figure S9: STEM (1), EDS Line scan (2), and mapping (3-4) for the samples 03Pt_01Cu_GD (A), 04Pt_01Cu_GD (B), and 06Pt_02Cu_GD (C).

Rietveld refinement provides detailed information on the average crystal structure, provided that the Bragg reflections are sufficiently well resolved. However, in nanomaterials, the reflections broaden substantially as the crystallite size decreases, becoming particularly pronounced for particle sizes below ~10 nm. [3] This broadening can preclude reliable structural refinement using conventional X-ray diffraction and Rietveld analysis. [3] In a scattering experiment, the measured intensity of a given phase scales with its weight fraction and the scattering power of its constituent atoms. In the present case, we aim to characterize Cu-doped Pt nanoparticles with sizes of approximately 10-20nm and a weight fraction of only 0.1%. Consequently, the scattering signal is expected to be overwhelmingly dominated by the $SrTiO_3$ support material, effectively masking the signal from the nanoparticles. As demonstrated in Figure S10a, no distinct signal corresponding to the Pt fcc structure is observed at the expected position of its main reflection (~2.7 $Å^{-1}$), precluding Rietveld refinement of the nanoparticle structure.

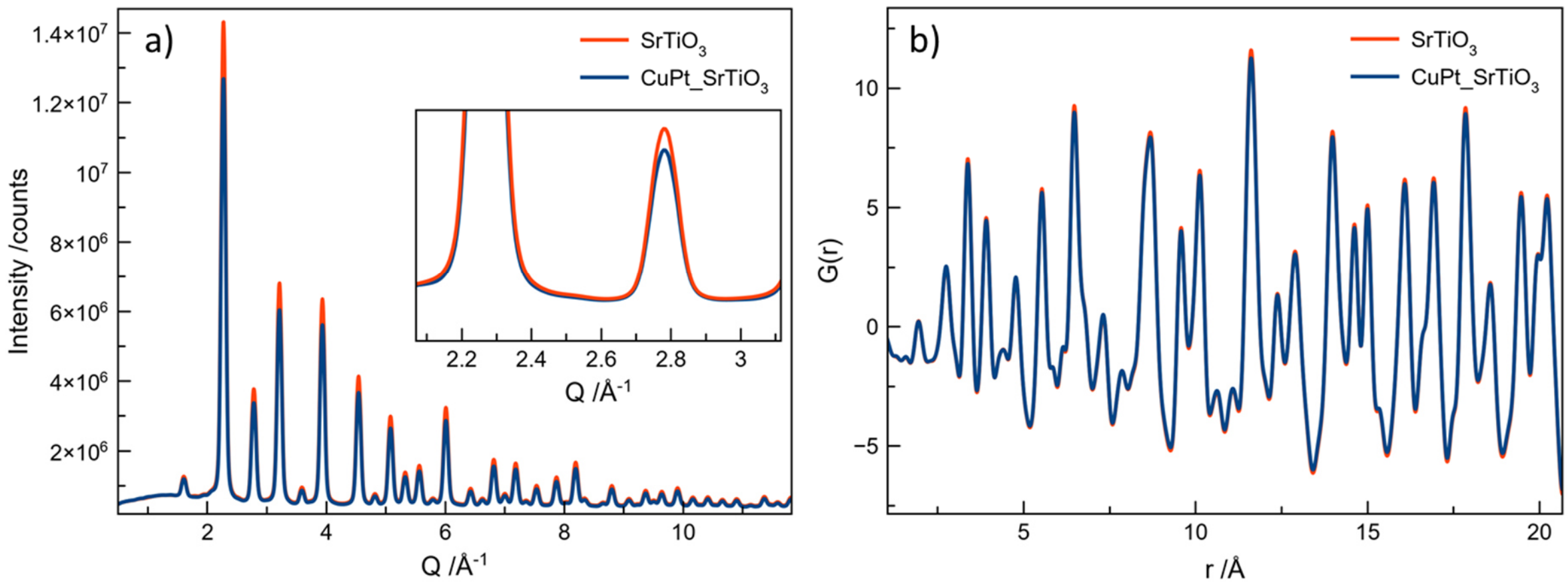

Figure S10: a) Total scattering data and b) corresponding pair distribution functions of untreated $SrTiO_3$ and 04Pt_01Cu_GD. The main reflection of Pt fcc is expected at ~2.7 $Å^{-1}$.

Fourier transformation of the total scattering (TS) data yields the pair distribution function (PDF),[4] which resembles atom-atom pair correlations in the material. However, due to the low scattering contribution of the Cu-doped Pt nanoparticles, their PDF signal cannot be distinguished from that of the $SrTiO_3$ support similar to the TS data as seen in Figure S10b. To overcome this limitation, we measured the TS data and generated PDFs of the bare support and of the support containing the Cu-doped Pt nanoparticles. Subtraction of these PDFs ($G(r)_{CuPt\text{-}SrTiO3}$-$G(r)_{SrTiO3}$) yields a differential PDF (d-PDF), which isolates the structural contribution arising from the difference between the two samples, i.e., the Cu-doped Pt nanoparticles.[4] In Figure S11a, the experimental d-PDF is compared with simulated PDFs for several Cu-Pt compositions and reference structures reported in the literature, including fcc Pt,[5] $CuPt_7$,[6] Cu_3Pt,[7,8] and fcc Cu [9]. Despite the very low fraction of the nanoparticle phase in the sample, distinct metal-metal correlation peaks at approximately 2.74, 4.73, 7.20, and 9.87 Å are clearly resolved in the d-PDF. The experimental peak positions show the closest agreement with the simulated $CuPt_7$ structure, as highlighted by the grey lines in Figure Sya. Based on this agreement, the CuPt7

structure was used as a model for fitting the experimental d-PDF over the range of 1-20 Å (Figure S11b). The refinement included the scale factor, lattice parameter a, Debye-Waller factors, and the spherical particle diameter (spdiameter). The latter refined to a value of 3.4 nm, in good agreement with the nanoparticle size determined by TEM.

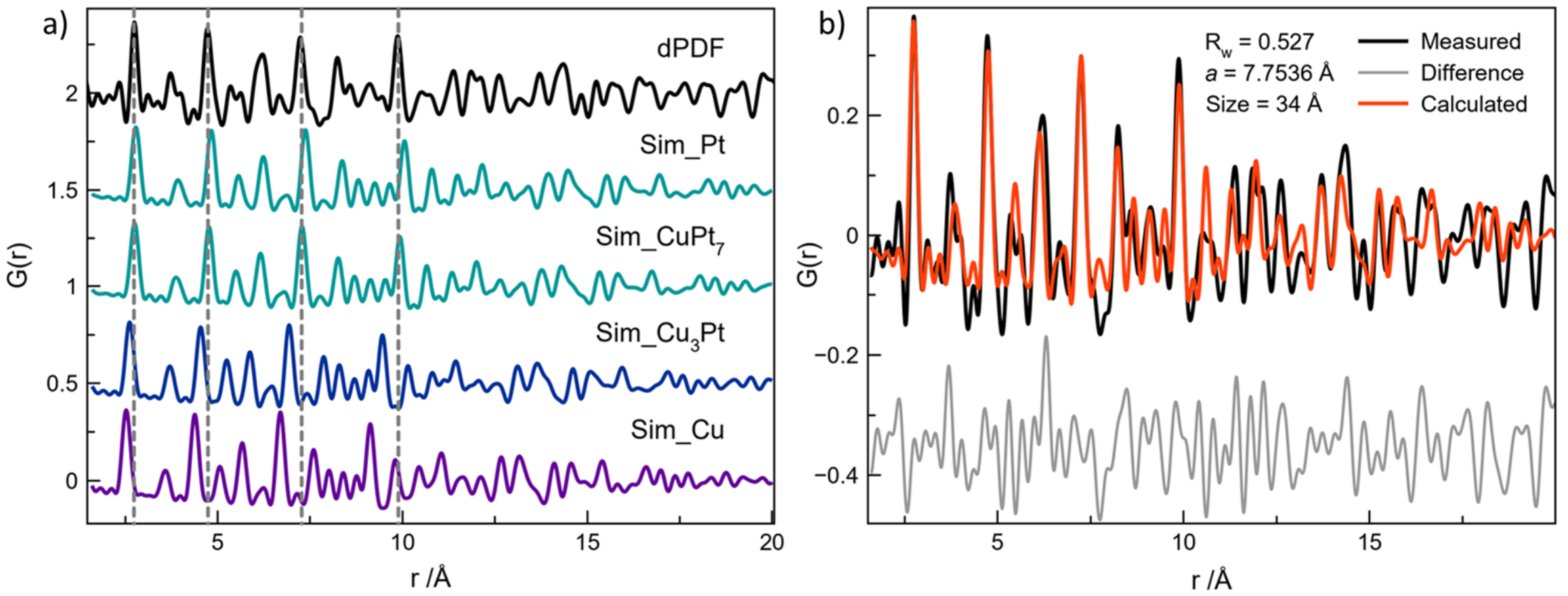

Figure S11: a) difference PDF ($G(r)_{CuPt\text{-}SrTiO3}$-$G(r)_{SrTiO3}$) and simulated PDFs of fcc Pt,[5] $CuPt_7$,[6] Cu_3Pt,[7,8] and Cu [9] b) Fitted dPDF using the CuPt7 structural model.

From our analysis, the nearest-neighbor Pt-Pt distance is determined to be 2.74 Å, compared with 2.79 Å for fcc Pt. The shorter distance indicates a contraction of the Pt lattice relative to pure Pt, suggesting that Cu is incorporated into the structure, consistent with the smaller atomic radius of Cu compared with Pt.

To estimate the relative Cu and Pt contents, we plotted the lattice parameter *a* of reported Cu-Pt phases with different Cu concentrations as a function of their relative Cu content (Figure S12). According to Vegard’s law,[3,10] a linear relationship between composition and lattice parameter is expected for substitutional solid solutions. A linear function was therefore fitted to the available reference data (blue squares in Figure S12), yielding $y = -0.0034x + 3.9443$. Using the experimentally determined lattice parameter of our sample (red circle in Figure S12), the alloy composition is estimated to be approximately 20 at.% Cu and 80 at.% Pt which is in great agreement with the relative intensity of Pt and Cu from the EDS line scan.

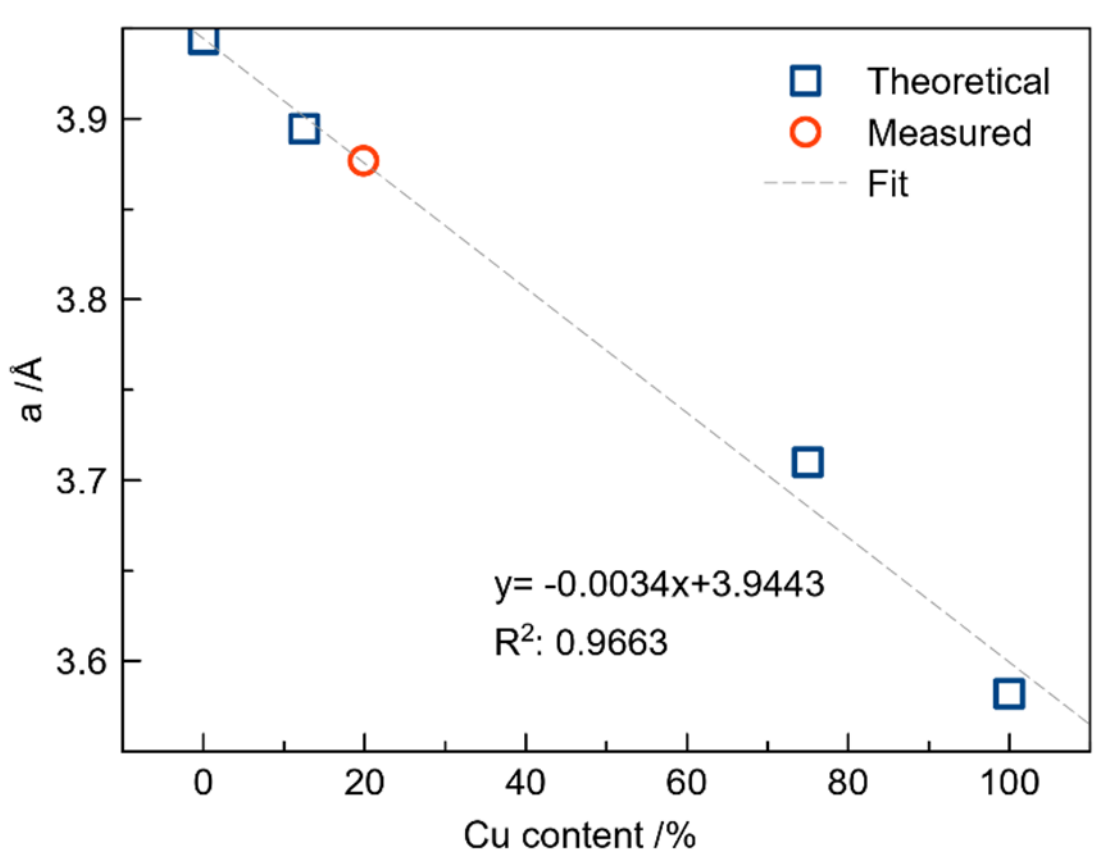

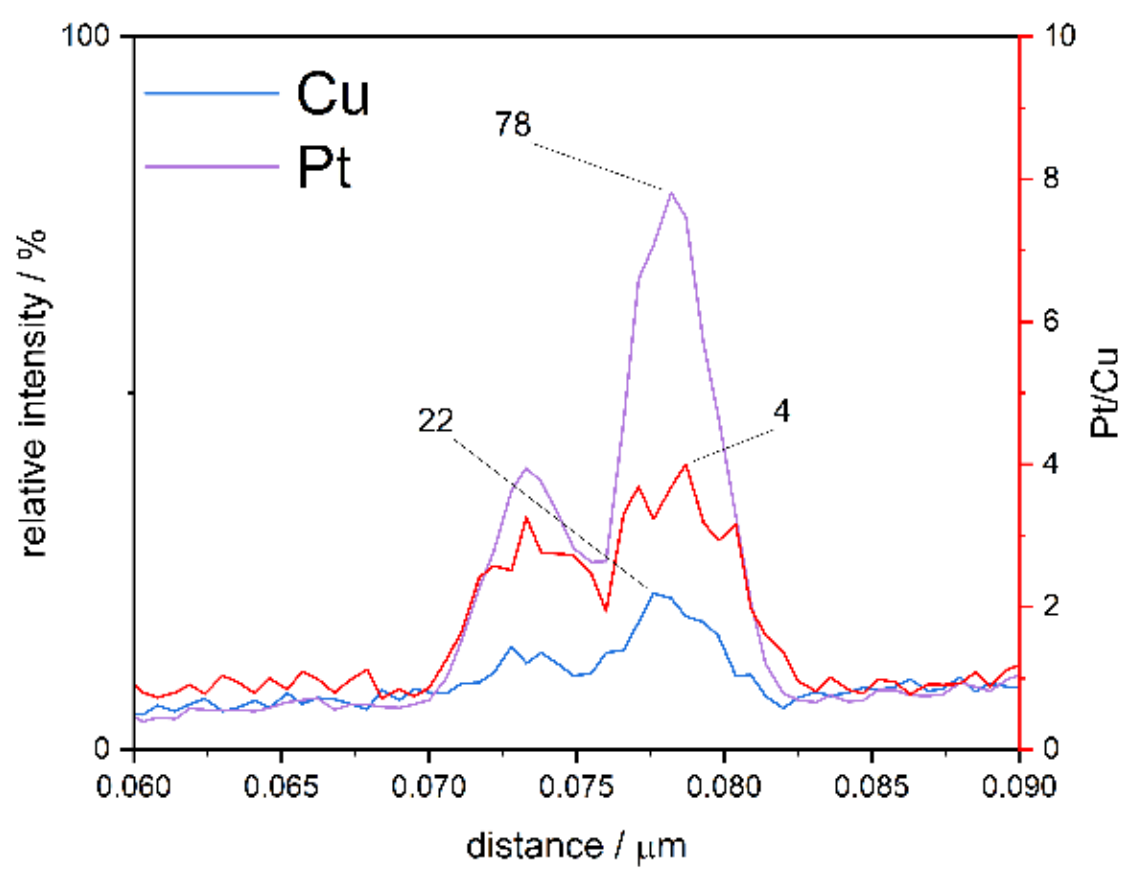

Figure S12: left: Lattice parameters as a function of Cu content of fcc Pt, $CuPt_7$, Cu_3Pt, and fcc Cu (blue squares) and sample 04Pt_01Cu_GD (red circle). Right: Relative intensity and Pt/Cu ratio from the EDS line scan of the 04Pt_01Cu_GD photocatalyst.

Of note, the specific ordering of Cu and Pt atoms in the $CuPt_7$ structure results in a doubling of the unit cell. Therefore, the refined lattice parameter was divided by two to enable direct comparison with the other structures. As a cross-check, the d-PDF was additionally refined using a simple fcc Pt structure model. This refinement yielded the same lattice parameter (a = 3.8769 Å, red circle in Figure S12), along with comparable Debye-Waller factors and spdiameter, and a similar Rwp value as to the fit with the $CuPt_7$ structure model.

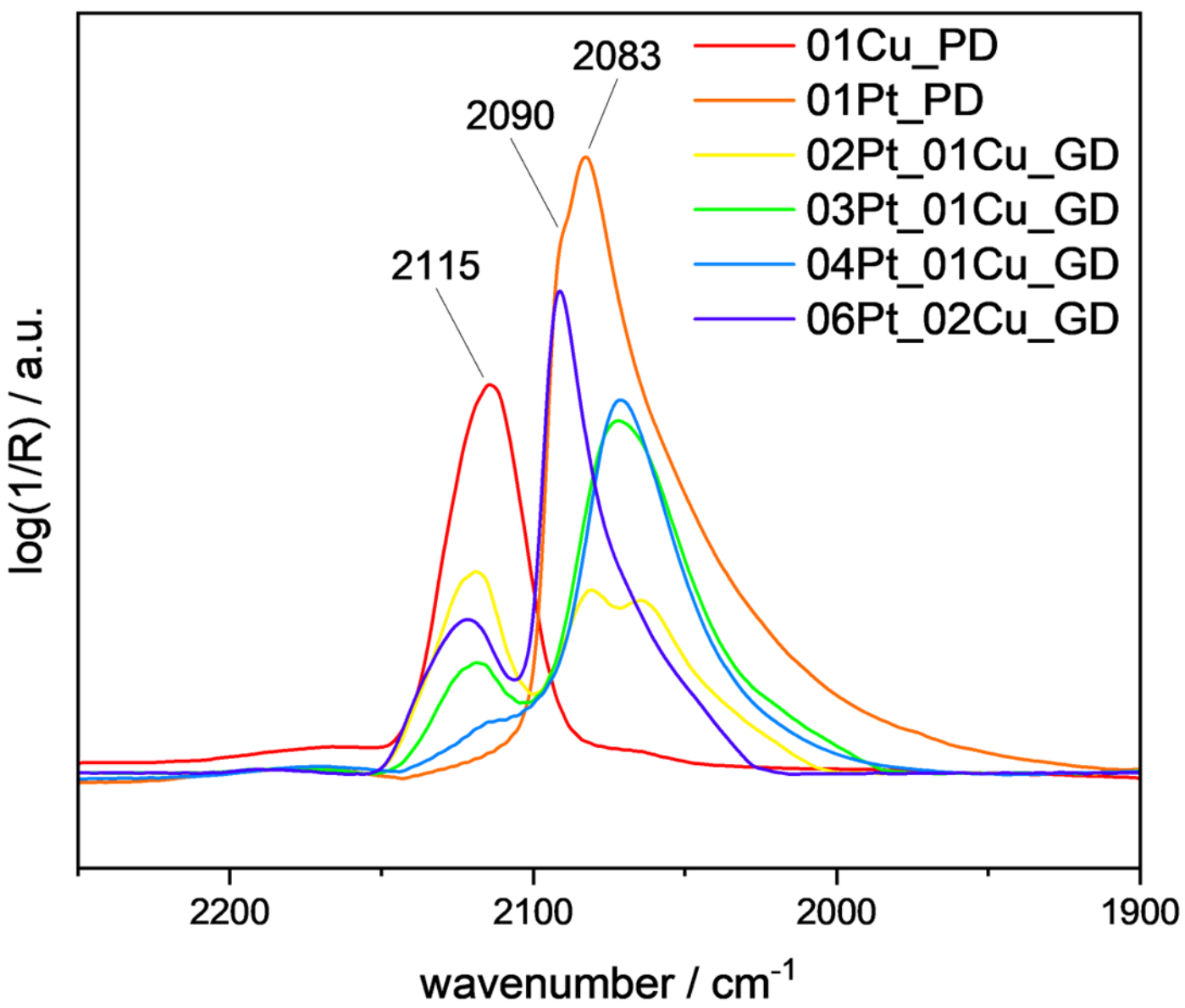

Figure S13: DRIFT spectra during CO adsorption on different samples after 15 min of adsorption of 1000 ppm CO.

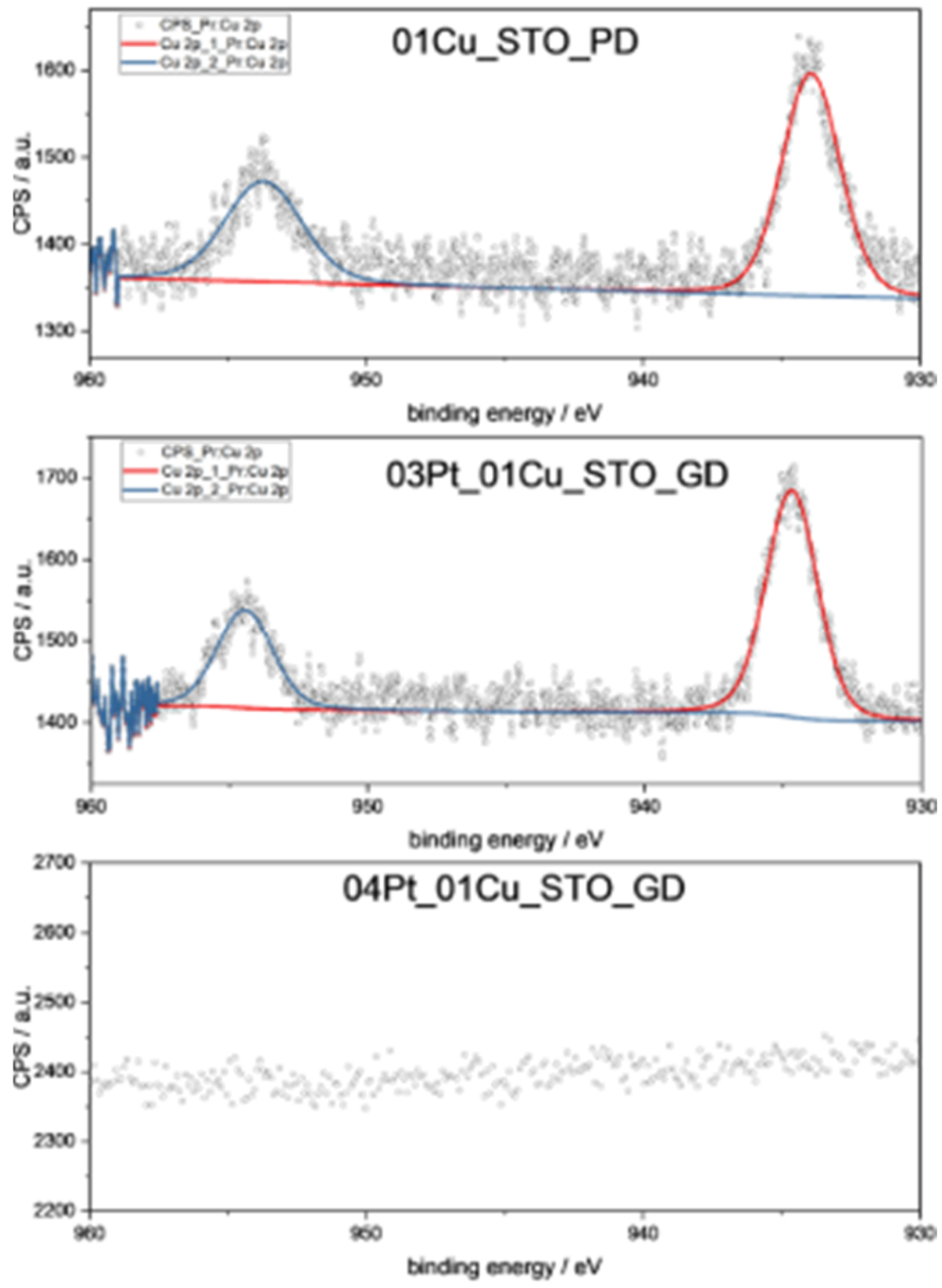

Figure S14: XPS spectra of the Cu 2p region.

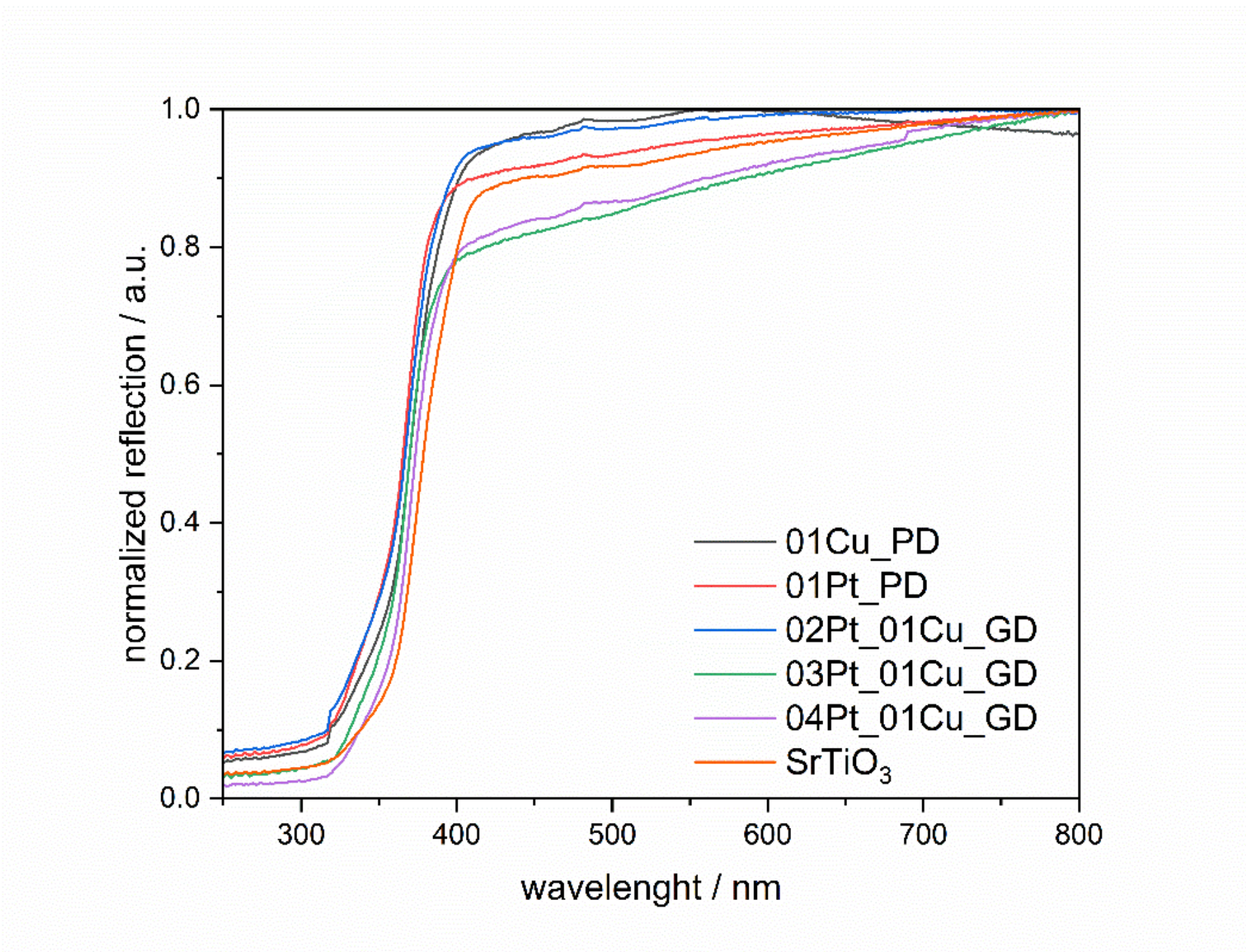

Figure S15: Diffuse reflectance ultraviolet-visible spectra of the different photocatalysts.

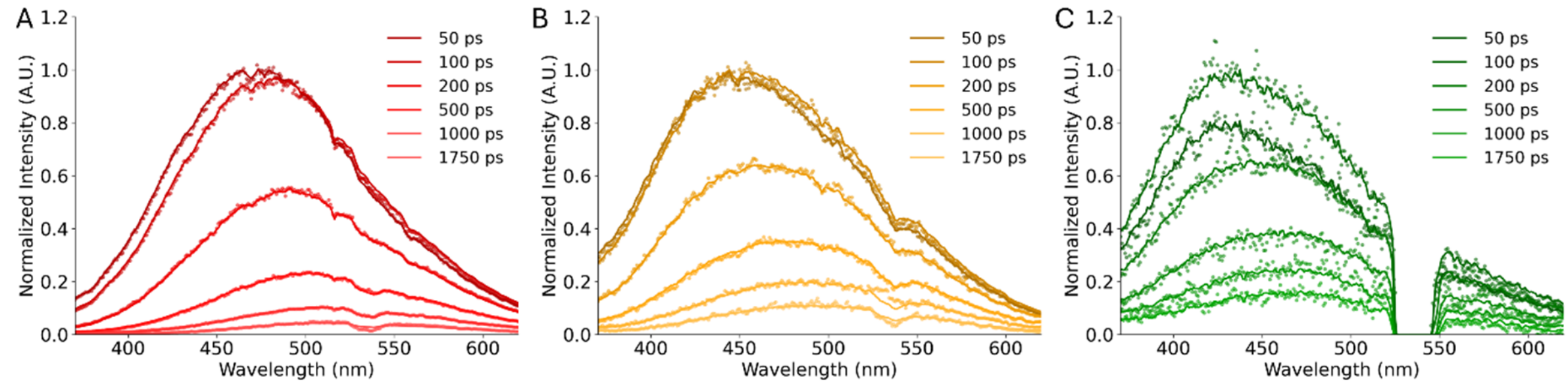

Figure S16: Spectra at various timepoints for 01Pt_PD (A), 03Pt_01Cu_GD (B) and 04pt_01Cu_GD (C) samples. Points are the raw data and lines the fits obtained from global analysis. The dip at 530-550 nm is due to residual scattering of the excitation laser.

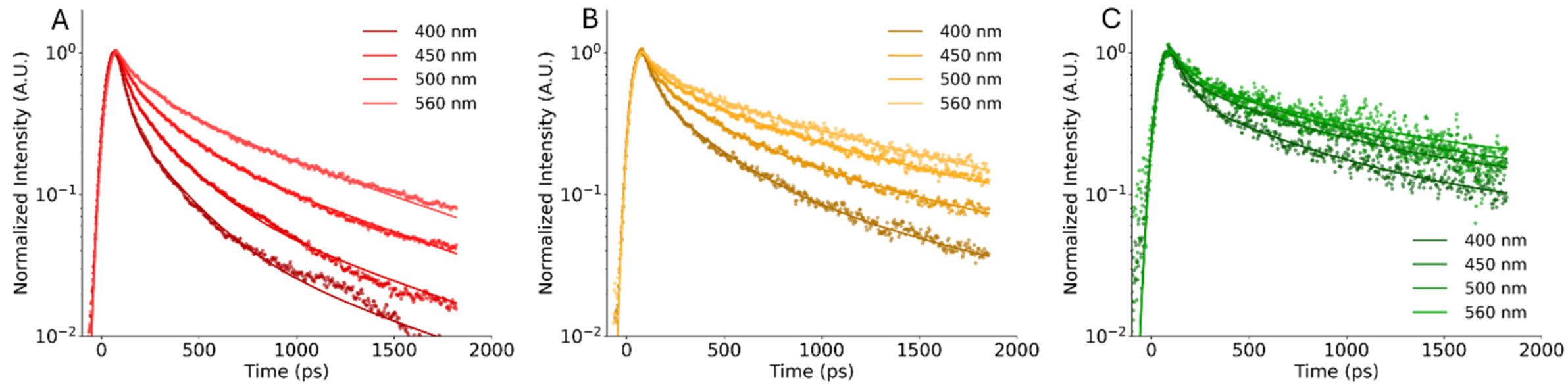

Figure S17: Time traces at various wavelengths 01Pt_PD (A), 03Pt_01Cu_GD (B) and 04pt_01Cu_GD (C) samples. Points are the raw data and lines the fits obtained from global analysis. The dip at 530-550 nm is due to residual scattering of the excitation laser

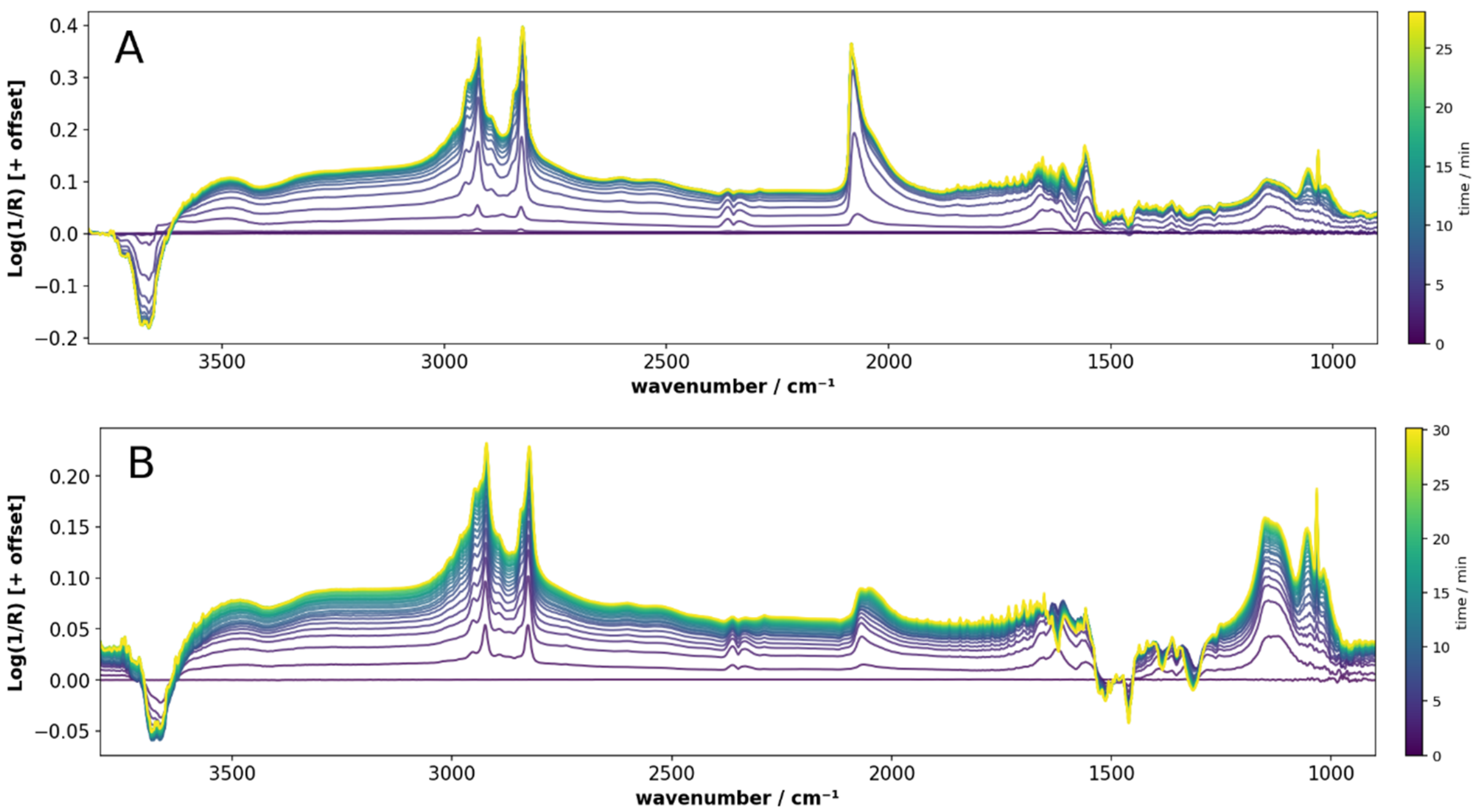

Figure S18: DRIFTS spectra for 01Pt_PD (A) und 04Pt_01Cu_GD (B) in 1.5% MeOH and 4.5% Water in 10 mL/min He at 100°C with. Background taken after H_2O cleaning procedure.

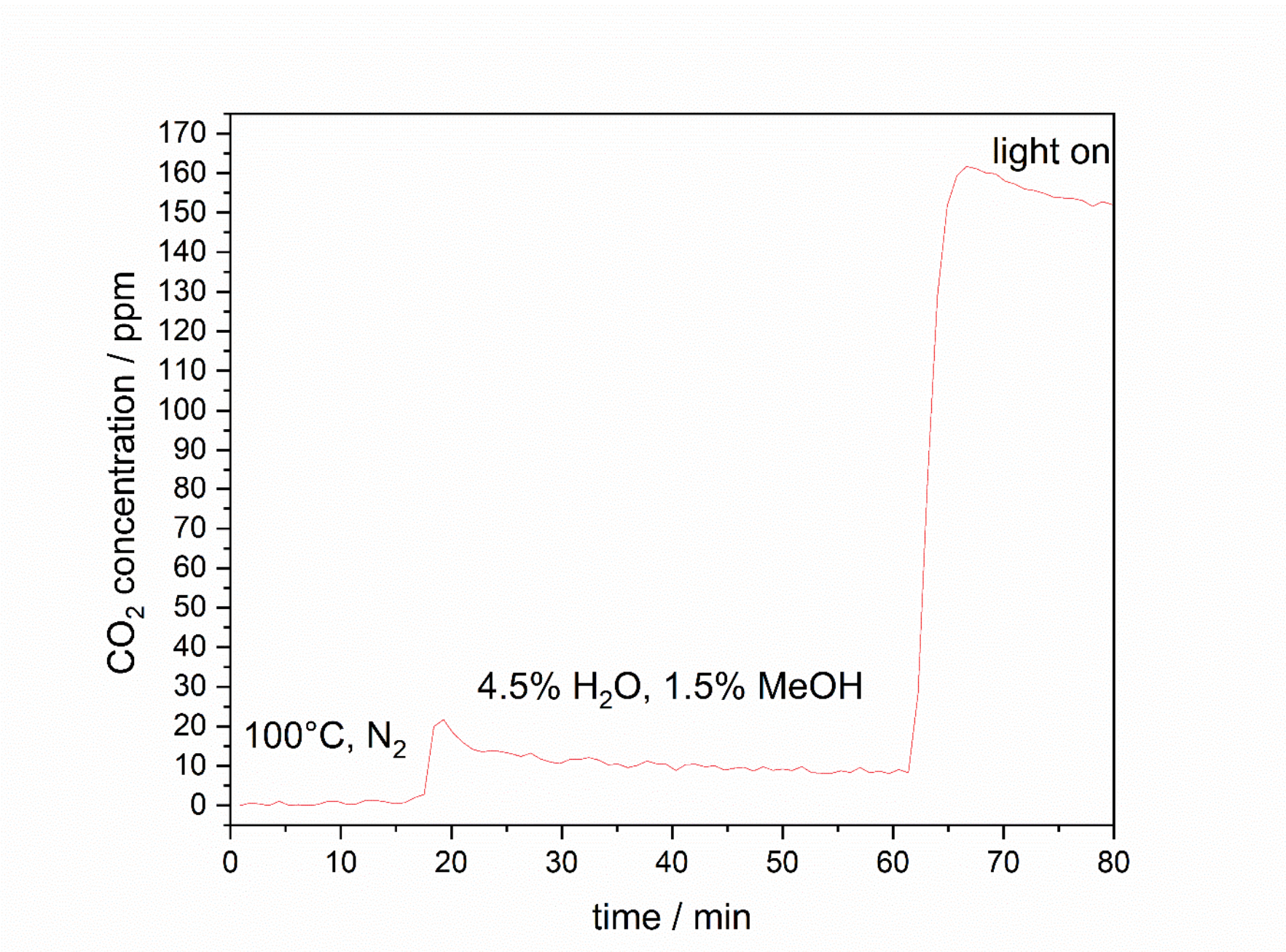

Figure S19: CO_2 formation prior to photocatalytic testing of 04Pt_01Cu_GD under dark conditions.

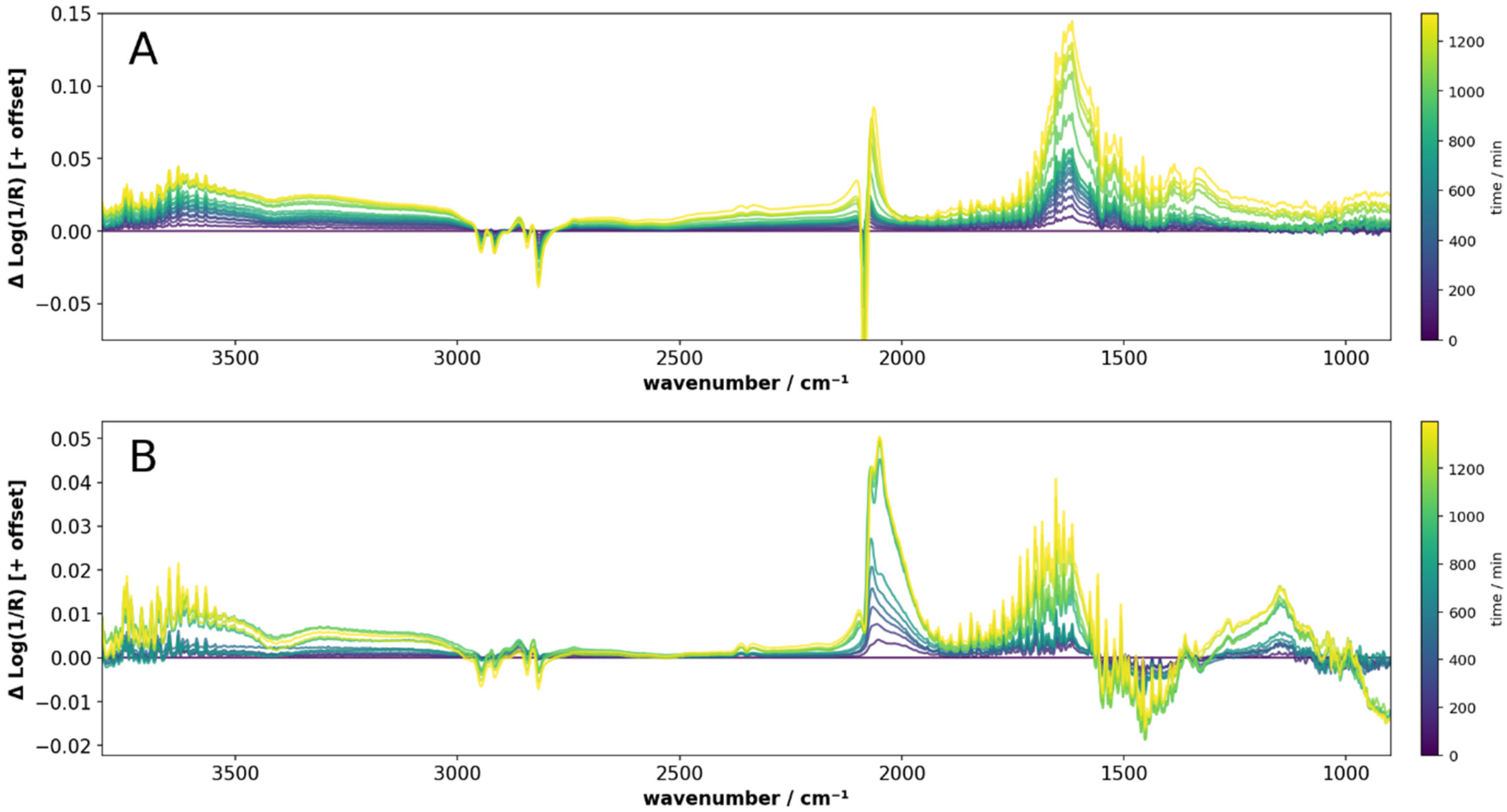

Figure S20: Difference DRIFTS spectra for 01Pt_PD (A) und 04Pt_01Cu_GD (B) during in photocatalytic MeOH dehydrogenation at 100°C with 1.5% MeOH and 4.5% Water in 10 mL/min He. Background taken after 30 minutes in reaction atmosphere at 100°C in the dark.

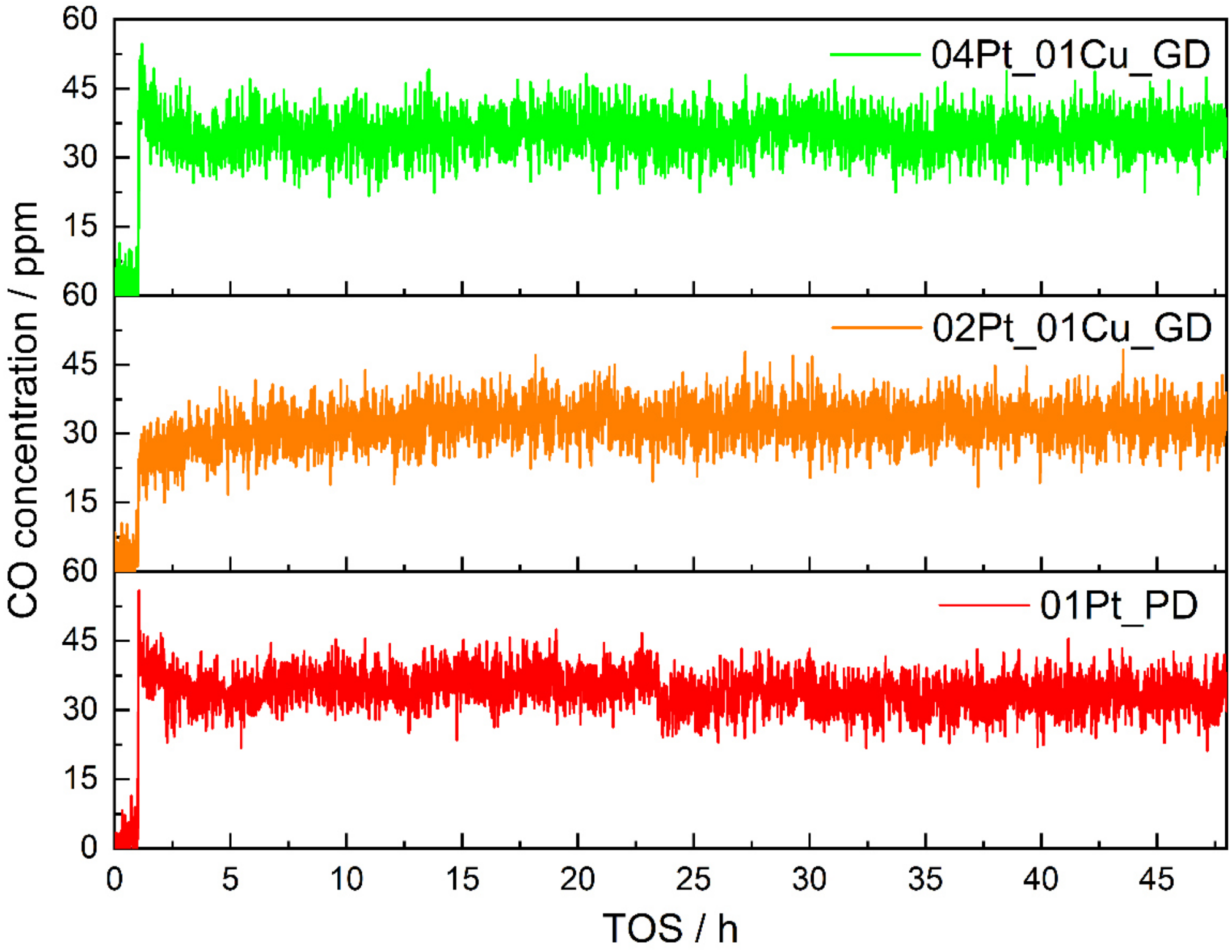

Figure S21: CO concentration during photocatalytic methanol dehydrogenation obtained in the gas-phase reactor set-up at 100°C for 01Pt_PD, 02Pt_01Cu_GD, and 04Pt_01Cu_GD.

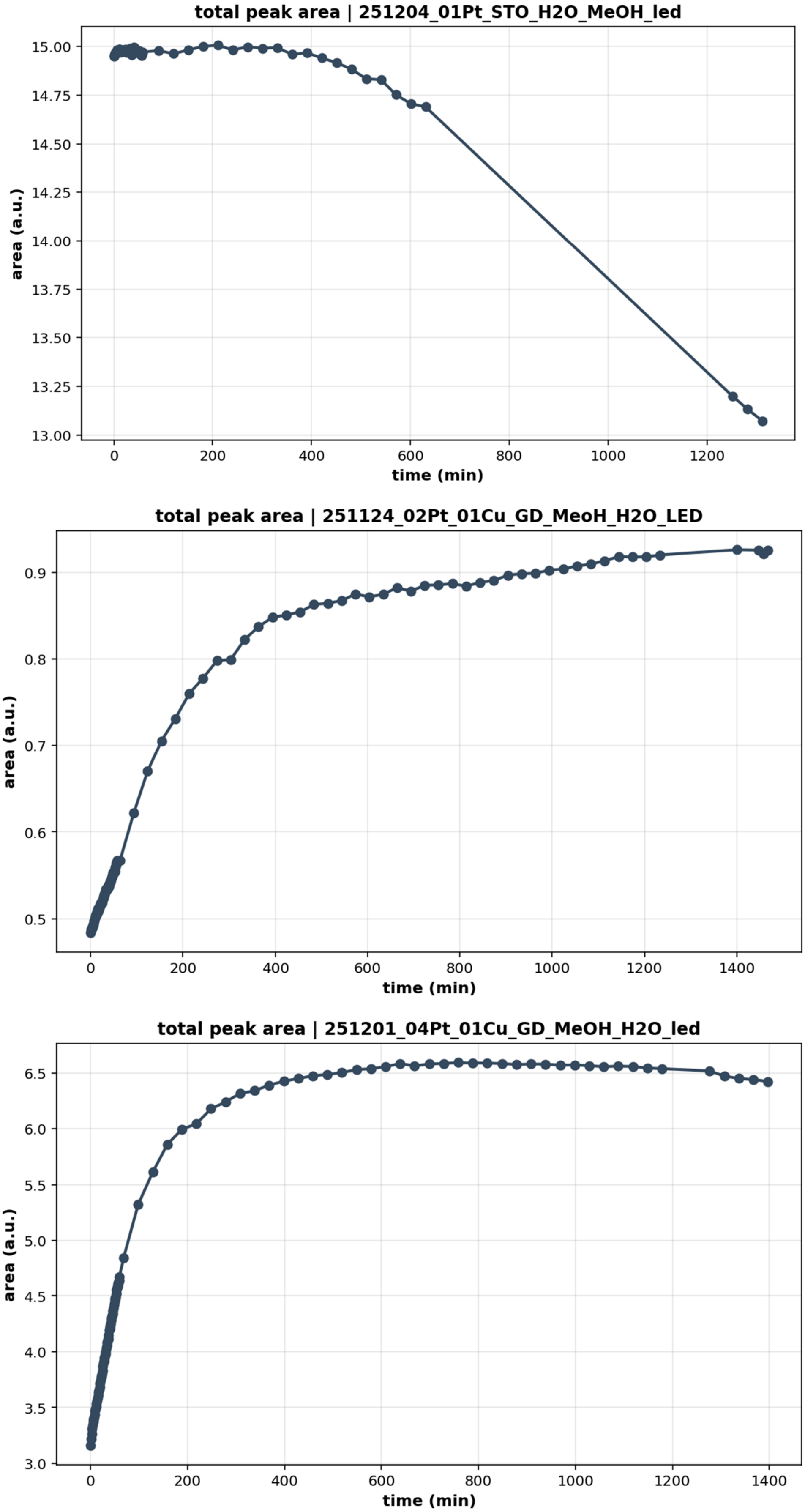

Figure S22: Trend of the total peak areas during photocatalytic methanol dehydrogenation in the in-situ DRIFTS cell for 01Pt_PD, 02Pt_01Cu_GD, and 04Pt_01Cu_GD.

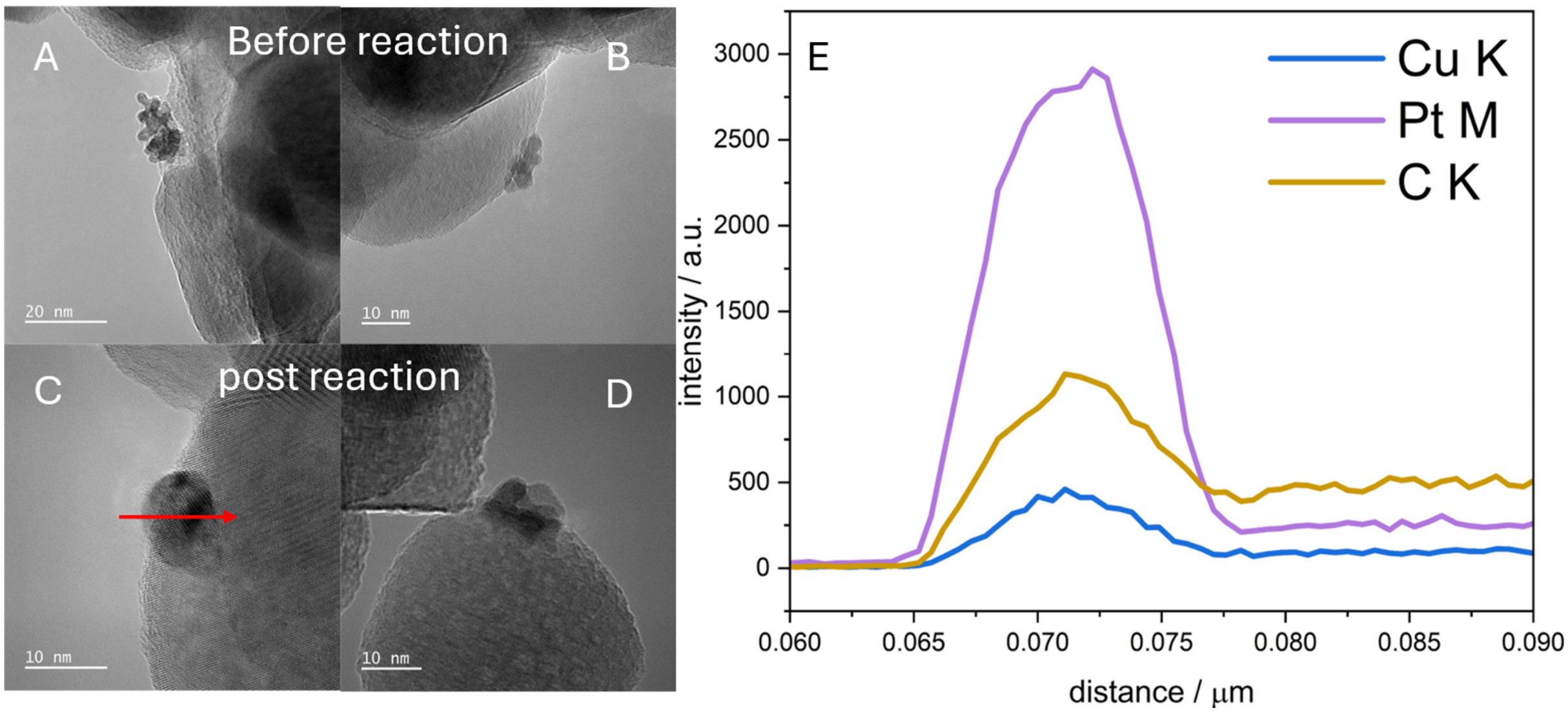

Figure S23: 04Pt_01Cu_GD TEM pre (A-B)/post (C-D) 48 h TOS; EDS line scan (E). Hexagonal Pt vanishes; smooth core–shell Pt NPs emerge. Elevated C indicates stabilizing adsorbates.

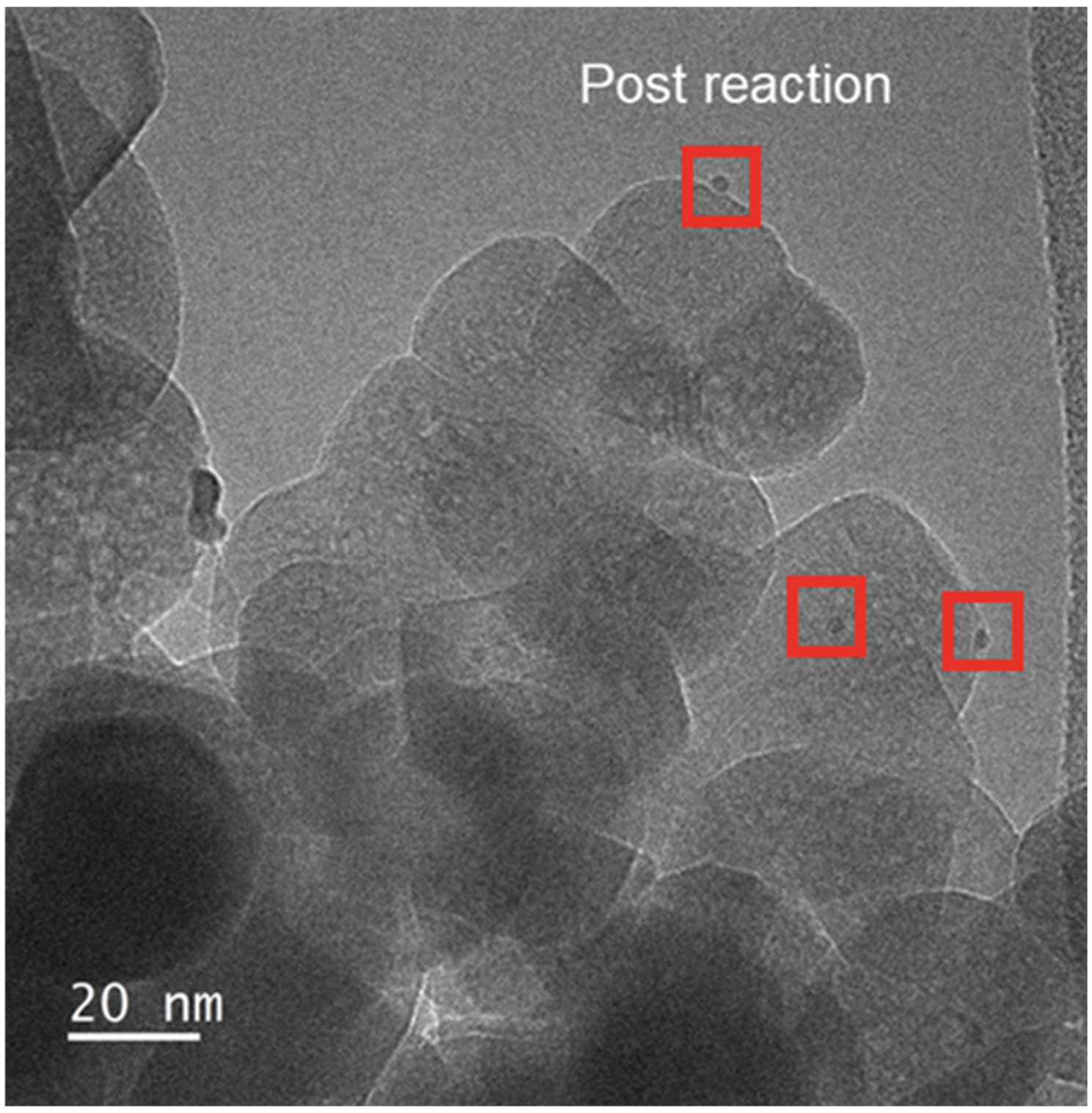

Figure S24: TEM images indicating the formation of small Pt NP post reaction for 04Pt_01Cu_GD.

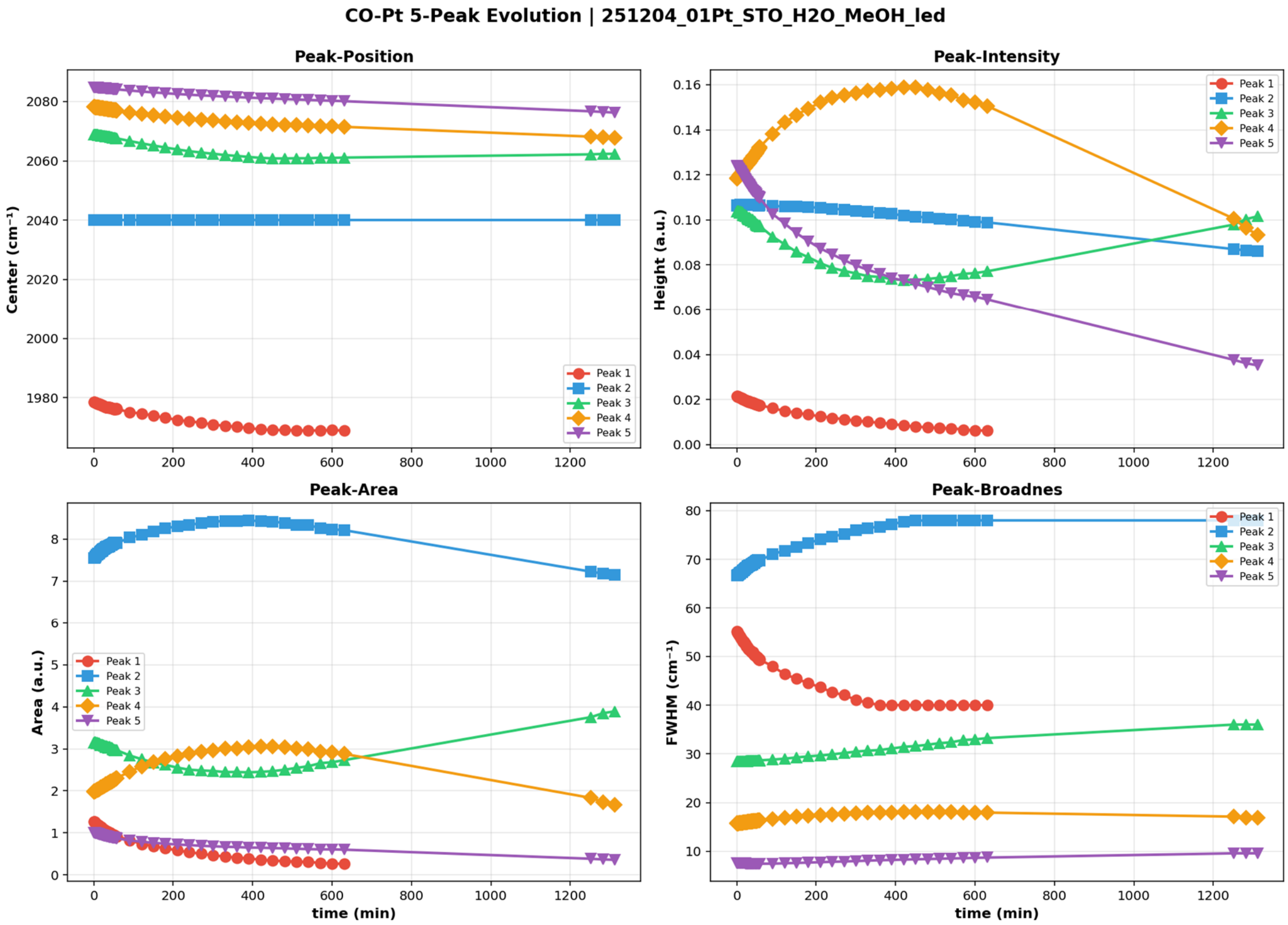

Figure S25: Temporal evolution of the contribution of different Pt-CO species (deconvoluted) during photocatalytic methanol dehydrogenation over 01Pt_PD..

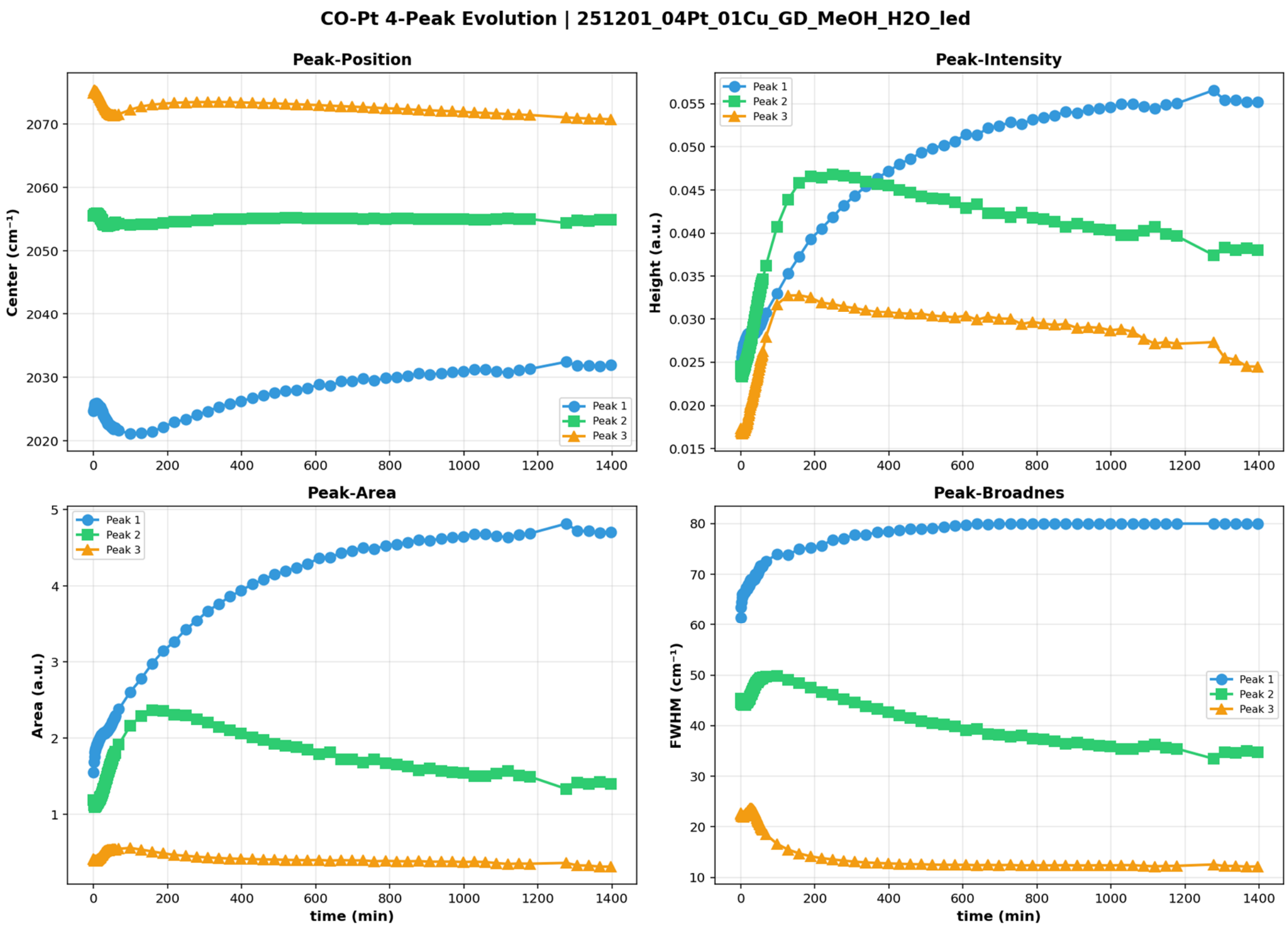

Figure S26: Temporal evolution of the contribution of different Pt-CO species (deconvoluted) during photocatalytic methanol dehydrogenation over 04Pt_01Cu_PD.

Calculation of the apparent quantum yield:

$$A = 60\ cm^2$$

$$I = 568\ \frac{W}{cm^2}$$

$$\lambda = 365\ nm$$

$$N_P = \frac{I\lambda}{hc} = 1.08 * 10^{21}\ \frac{1}{sm^2}$$

$$n_P = \frac{AN_P}{N_A} = 3.87 * 10^4\ \frac{\mu mol}{s}$$

$$AQY_{FA} = \frac{2h^{+}n(FA)}{n_P}$$

A = illuminated Area, I = light intensity, λ = wavelength, h = plancks constant, c = speed of light, N_a = Avogadro's number, N_P = total photon flux, n_P = molar photon flux, n(X) = evolution rate

Note that the wavelength of the used LEDs is a spectrum and not a single wavelength. Therefore, the photon flux is an approximation but since the approximation is constant for all samples a comparison within our results is still feasible.

References

[1] R. Brüninghoff, K. Wenderich, J.P. Korterik, B.T. Mei, G. Mul, A. Huijser, J. Phys. Chem. C 123 (2019) 26653–26661.

[2] I.H.M. van Stokkum, J. Weißenborn, S. Weigand, J.J. Snellenburg, Photochemical & photobiological sciences Official journal of the European Photochemistry Association and the European Society for Photobiology 22 (2023) 2413–2431.

[3] C.F. Holder, R.E. Schaak, ACS nano 13 (2019) 7359–7365.

[4] T.L. Christiansen, S.R. Cooper, K.M.Ø. Jensen, Nanoscale advances 2 (2020) 2234–2254.

[5] G. Bredig, R. Allolio, Zeitschrift für Physikalische Chemie 126U (1927) 41–71.

[6] A.R. Cabral, R. Skála, A. Vymazalová, A. Kallistová, B. Lehmann, J. Jedwab, T. Sidorinová, Mineral. mag. 78 (2014) 739–745.

[7] VRML crystal structure gallery, 2010. Available from: http://jcrystal.com/steffenweber/gallery/StructureTypes/st4.html (08.18.2026).

[8] J. Zemann, Acta Cryst 18 (1965) 139.

[9] C. Li, D. Fernández González (Eds.), Copper - Properties, Metallurgy and Applications, IntechOpen, 2026.
[10] A.R. Denton, N.W. Ashcroft, Physical review. A, Atomic, molecular, and optical physics 43 (1991) 3161–3164.
